# Untangling the clinicopathological significance of MRE11-RAD50-NBS1 complex in sporadic breast cancers

**DOI:** 10.1038/s41523-021-00350-5

**Published:** 2021-11-15

**Authors:** Adel Alblihy, Ahmed Shoqafi, Michael S. Toss, Mashael Algethami, Anna E. Harris, Jennie N. Jeyapalan, Tarek Abdel-Fatah, Juliette Servante, Stephen Y. T. Chan, Andrew Green, Nigel P. Mongan, Emad A. Rakha, Srinivasan Madhusudan

**Affiliations:** 1 Nottingham Biodiscovery Institute, School of Medicine, University of Nottingham, University Park, Nottingham, NG7 3RD UK; 2 Medical Center, King Fahad Security College (KFSC), Riyadh, 11461 Saudi Arabia; 3 Department of Pathology, Nottingham University Hospitals, City Hospital Campus, Nottingham, NG5 1PB UK; 4 Department of Oncology, Nottingham University Hospitals, City Hospital Campus, Nottingham, NG5 1PB UK; 5 Department of Medicine, Royal Derby Hospital, Derby, DE22 3NE UK

**Keywords:** Breast cancer, Prognostic markers

## Abstract

The MRE11–RAD50–NBS1 (MRN) complex is critical for genomic stability. Although germline mutations in MRN may increase breast cancer susceptibility, such mutations are extremely rare. Here, we have conducted a comprehensive clinicopathological study of MRN in sporadic breast cancers. We have protein expression profiled for MRN and a panel of DNA repair factors involved in double-strand break repair (BRCA1, BRCA2, ATM, CHK2, ATR, Chk1, pChk1, RAD51, γH2AX, RPA1, RPA2, DNA-PKcs), RECQ DNA helicases (BLM, WRN, RECQ1, RECQL4, RECQ5), nucleotide excision repair (ERCC1) and base excision repair (SMUG1, APE1, FEN1, PARP1, XRCC1, Pol β) in 1650 clinical breast cancers. The prognostic significance of *MRE11*, *RAD50* and *NBS1* transcripts and their microRNA regulators (*hsa-miR-494* and *hsa-miR-99b*) were evaluated in large clinical datasets. Expression of MRN components was analysed in The Cancer Genome Atlas breast cancer cohort. We show that low nuclear MRN is linked to aggressive histopathological phenotypes such as high tumour grade, high mitotic index, oestrogen receptor- and high-risk Nottingham Prognostic Index. In univariate analysis, low nuclear MRE11 and low nuclear RAD50 were associated with poor survival. In multivariate analysis, low nuclear RAD50 remained independently linked with adverse clinical outcomes. Low RAD50 transcripts were also linked with reduced survival. In contrast, overexpression of *hsa-miR-494* and *hsa-miR-99b* microRNAs was associated with poor survival. We observed large-scale genome-wide alterations in MRN-deficient tumours contributing to aggressive behaviour. We conclude that MRN status may be a useful tool to stratify tumours for precision medicine strategies.

## Introduction

The MRE11–RAD50–NBS1 complex (MRN), a chemo-mechanical molecular machine, is critical for the maintenance of genomic stability^1–3^. MRN is required not only for processing DNA damage but also for oncogene-induced replication stress. MRN is a hexameric complex consisting of two RAD50 subunits (ATPase activity), two MRE11 subunits (DNA structure-specific endo- or exonuclease activity) and two NBS1 subunits (a regulatory docking protein with phosphopeptide-interacting forkhead-associated and BRCA1 C-terminal domains flexibly linked to an MRE11 interface and adjacent C-terminal ATM kinase interaction motif). The interaction of MRE11, RAD50 and NBS1 together promote MRN complex stability^1–3^.

MRN is recruited by RAD17 to sites of double-strand breaks (DSBs), which activates it^4^. MRN activates ataxia-telangiectasia-mutated (ATM) kinase, which in turn phosphorylates more than 700 proteins. ATM-induced CHK2 phosphorylation coordinates checkpoint signalling^5,6^. During homologous recombination repair (HR), MRN initiates the 5′ resection, which is followed by further resection by EXO1 or DNA2 nuclease. The resulting 3′ single-strand DNA (ssDNA) overhangs are loaded with replication protein A (RPA). Ataxia-telangiectasia and RAD3-related (ATR) is then recruited to RPA-coated ssDNA. Activated ATR, in turn, phosphorylates CHK1, which contributes to cell cycle regulation. During HR, the BRCA2–RAD51 complex nucleates the formation of RAD51 nucleofilaments and later displaces RPA from ssDNA. RAD51 nucleofilaments then promote homology search and strand invasion in association with BRCA1–BARD1 for error-free repair through the resolution of repair intermediates. Although classical NHEJ does not require MRN, microhomology-mediated end-joining operates through MRN–CtIP-mediated resection, followed by priming based on microhomology, flap removal and gap filling^1–3^.

Germline mutations in MRE11, NBS1 or RAD50 can cause genomic instability syndromes characterized by immunodeficiency, hypersensitivity to radiation and cancer predisposition^7,8^. Mutations in the MRE11 cause ataxia-telangiectasia-like disorder^9^. Mutation in NBS1 can cause Nijmegen breakage syndrome (NBS)^7^. RAD50 deficiency has also been reported in a case of NBS-like disorder^8^. Polymorphic variation in *MRE11*, *RAD50* and *NBS1* genes may increase cancer risk including breast cancer predisposition^10–12^. However, the clinicopathological significance of MRN in human sporadic breast cancers has not been clearly defined. In the current study, we have used immunohistochemistry (IHC) to examine the expression of MRN and other factors involved in DSB repair (BRCA1, BRCA2, ATM, CHK2, ATR, CHK1, pCHK1, RAD51, γH2AX, RPA1, RPA2, DNA-PKcs), RECQ helicases (BLM, WRN, RECQ1, RECQL4, RECQ5), nucleotide excision repair (ERCC1) and base excision repair (SMUG1, APE1, FEN1, PARP1, XRCC1, Pol β) in a large clinical cohort of 1650 breast cancers. Detailed bioinformatics of MRN interactors at the transcriptomic level as well as the prognostic value of *MRE11* mRNA, *RAD50* mRNA, *NBS1* mRNA expressions and their microRNA (miRNA) regulators (*hsa-miR-494* and *hsa-miR-99b*) were evaluated in large clinical datasets.

## Results

### MRN and histopathological features

**Fig. 1:**
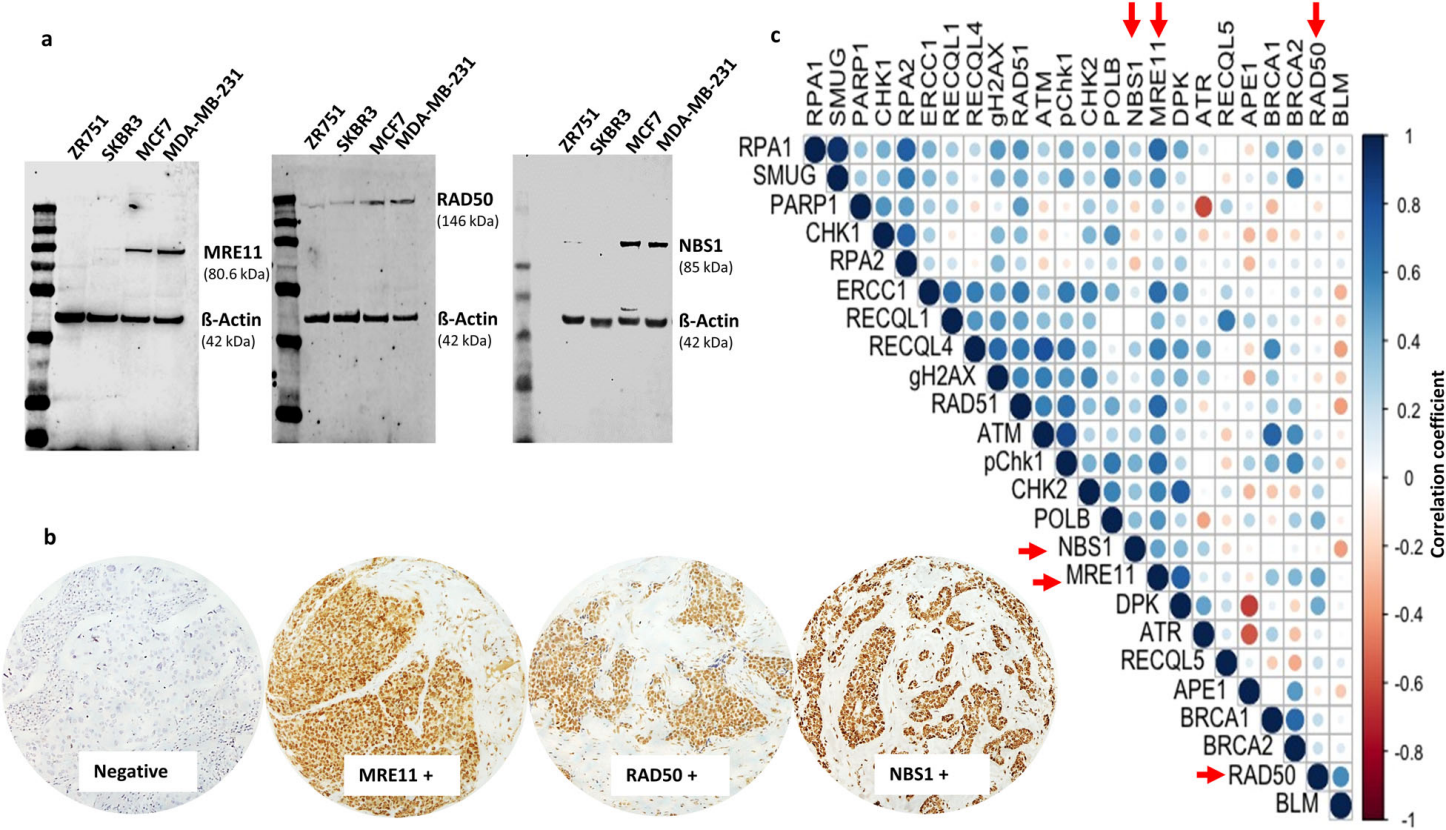
MRN expression in breast cancers. **a** Western blot showing MRE11, RAD50 and NBS1 protein expression in a panel of breast cancer cell lines. All blots derive from the same experiment and they were processed in parallel. **b** Photomicrographs showing immunohistochemical staining of MRE11, RAD50 and NBS1 in breast cancers. **c** Correlation Matrix showing the correlation between levels of MRE11, NBS1 and RAD50 protein expressions and other DNA repair biomarkers. Blue colour refers to positive (+) correlation, while the red colour reflects negative (−) correlations. The size of the circles and intensity of the colour propionate to the correlation coefficient. The image was generated using RStudio software. Red arrow = proteins investigated in the current study i.e. MRE11, RAD50 and NBS1.

We initially tested MRE11, RAD50 and NBS1 protein expression in a panel of breast cancer cell lines [MCF-7 (ER+, luminal A), ZR-75-1 (ER+, luminal B), SKBR3 (HER2+), MDA-MB-231 (triple-negative)]. As shown in Fig. 1a, MCF-7 and MDA-MB-231 showed robust expression of MRE11, RAD50 and NBS1. On the other hand, ZR-75-1 and SKBR3 had low MRE11, RAD50 and NBS1 protein expression. We proceeded to an immunohistochemical evaluation in a clinic cohort of 1650 breast cancers.

We observed nuclear and cytoplasmic MRE11 expression in breast cancers (Fig. 1b). As the nuclear level of MRE11 primarily contributes to DDR function, we first evaluated nuclear MRE11 and correlated to histopathological features. The data are summarized in Table 1. Low nuclear MRE11 [46% (302/659)] was strongly associated with aggressive clinicopathological features including high tumour grade, high mitotic index, de-differentiation, marked pleomorphism, HER2+, oestrogen receptor (ER)- and high-risk Nottingham Prognostic Index (NPI) phenotypes (all *p* < 0.01). Cytoplasmic staining of MRE11 [54% (357/659)] was associated with low-grade tumours (*p* = 0.01) with reduced pleomorphism (*p* = 0.01) (Supplementary Table 3).

**Table 1:** MRN complex protein expression and clinicopathological features in breast cancer.

	MRE11 (nuclear) expression			RAD50 (nuclear) expression			NBS1 (nuclear) expression		
	Low	High	*P* value	Low	High	*P* value	Low	High	*P* value
Variable	*N* (%)	*N* (%)		*N* (%)	*N* (%)		*N* (%)	*N* (%)	
Tumour size									
≤2 cm	150 (42)	204 (58)	0.055	165 (43)	217(57)	0.084	223 (44)	287 (56)	**0.004**
>2 cm	152 (49.8)	153 (50.2)		174 (50)	177 (50)		342 (52)	314 (48)	
Nottingham Prognostic Index (NPI)									
1	64 (18)	112 (37)		84 (58)	11 9 (34)		131 (26)	230 (35)	
2	217 (61)	155 (51)	**<0.001**	228 (60)	184 (52)	**0.001**	285 (56)	327 (50)	**0.002**
3	73 (21)	38 (12)		70 (18)	48 (14)		94 (18)	99 (15)	
Tumour grade									
G1	27 (8)	60 (20)	**<0.001**	45 (12)	61 (17)	**<0.001**	75 (15)	120 (18)	**<0.001**
G2	100 (28)	127 (42)		112 (29)	146 (42)		148 (29)	251 (38)	
G3	226 (64)	118 (39)		223 (59)	144 (41)		288 (56)	284 (44)	
Mitotic index									
M1 (low; mitoses < 10)	71 (21)	126 (43)	**<0.001**	88 (24)	144 (42)	**<0.001**	152 (31)	240 (38)	**0.002**
M2 (medium; mitoses 10–18)	72 (21)	66 (22)		80 (22)	71 (21)		85 (17)	131 (21)	
M3 (high; mitosis > 18)	199 (58)	102 (35)		198 (54)	125 (37)		256 (52)	260 (41)	
Tubule formation									
1 (>75% of definite tubule)	10 (3)	16 (5)	**0.004**	11 (3)	20 (6)	0.107	32 (7)	33 (5)	0.547
2 (10–75% definite tubule)	97 (28)	113 (38)		120 (33)	120 (35)		164 (33)	224 (36)	
3 (<10% definite tubule)	235 (69)	165 (56)		235 (64)	200 (59)		197 (60)	374 (59)	
Pleomorphism									
1 (small-regular uniform)	2 (0.6)	12 (4)	**<0.001**	5 (1)	9 (3)	0.063	9 (2)	19 (3)	**<0.001**
2 (moderate variation)	95 (30)	128 (44)		121 (33)	135 (40)		154 (31)	277 (44)	
3 (marked variation)	244 (72)	154 (52)		240 (66)	195 (57)		329 (67)	334 (53)	
Her2 overexpression									
No	273 (80)	70 (20)	**<0.001**	310 (84)	61 (16)	0.077	415 (86)	67 (14)	0.525
Yes	269 (91)	28 (9)		299 (88)	40 (12)		548 (87)	79 (13)	
ER/PR expression status									
ER−/PR−	114 (33)	45 (15)	**<0.001**	110 (30)	63 (19)	**0.003**	148 (30)	121 (19)	**<0.001**
ER−/PR+	0 (0)	0 (0)		0 (0)	1 (0)		0 (0)	1 (0)	
ER+/PR−	56 (16)	47 (16)		64 (17)	54 (16)		80 (16)	115 (18)	
ER+/PR+	175 (51)	204 (69)		199 (53)	219 (65)		260 (53)	395 (63)	

Bold values indicate statistical significance *p* values.

We also observed nuclear and cytoplasmic staining for NBS1 (Fig. 1b). Low nuclear NBS1 [48% (565/1166)] was associated with larger tumours, higher grade, high mitotic index, marked pleomorphism, ER- and high-risk NPI phenotypes (all *p* < 0.01). Low cytoplasmic NBS1 [48% (565/1166)] was associated only with de-differentiation (*p* < 0.001) (Supplementary Table 3).

We observed nuclear-only staining for *RAD50* expression (Fig. 1b). Low nuclear RAD50 [46% (339/733)] was highly associated with high tumour grade, high mitotic index, ER- and high-risk NPI phenotypes (all *p* values < 0.01) (Table 1).

Taken together, the data suggest that low MRN protein levels may contribute to aggressive histopathological features in breast cancer.

### MRN and correlation to other DNA repair proteins

Given the critical role played by MRN in DDR and the interaction with several DNA repair proteins in multiple pathways^1–3^, we correlated MRN expression with other proteins involved in DSB repair (BRCA1, BRCA2, ATM, CHK2, ATR, Chk1, pChk1, RAD51, γH2AX, RPA1, RPA2, DNA-PKcs), RECQ helicases (BLM, WRN, RECQ1, RECQL4, RECQ5), nucleotide excision repair (ERCC1) and base excision repair (SMUG1, APE1, FEN1, PARP1, XRCC1, Pol β) in the breast cancer cohort. The data are summarized in Supplementary Table 4. We observed a strong association between MRE11 and several DNA repair proteins (Fig. 1c), including RAD50, NBS1, BCRA1, ATM, CHK2, CHK1, pCHK1, RAD50, RAD51, γH2AX, RPA1, DNA-PKcs, BLM, RECQL1, RECQ4, RECQ5, ERCC1, SMUG1 and POLβ (all *p* values < 0.05, full data shown in Supplementary Tables 4 and 5 and Supplementary Data 1). A significant association between RAD50 and NBS1, BCRA1, CHK2, ATR, CHK1, pCHK1, RAD51, γH2AX, RPA1, RPA2, DNA-PKcs, BLM, RECQL1, RECQ4, RECQ5, ERCC1, SMUG1, PARP1 and POLβ was also evident (all *p* values < 0.05, full data shown in Supplementary Tables 4 and 5 and Supplementary Data 1). Similarly, significant association between NBS1 and BCRA1, ATM, CHK2, CHK1, pCHK1, RAD51, γH2AX, RPA1, DNA-PKcs, BLM, RECQL1, RECQ5, SMUG and POLβ was observed (all *p* values < 0.05, full data shown in Supplementary Tables 4 and 5 and Supplementary Data 1).

Together, the data provide evidence that low nuclear MRN is associated with reduction in the level of other key DNA repair proteins in a proportion of breast cancers, which together could contribute to aggressive histopathological phenotypes. We proceeded to evaluate the prognostic significance of MRN in breast cancer.

### MRN and survival

**Fig. 2:**
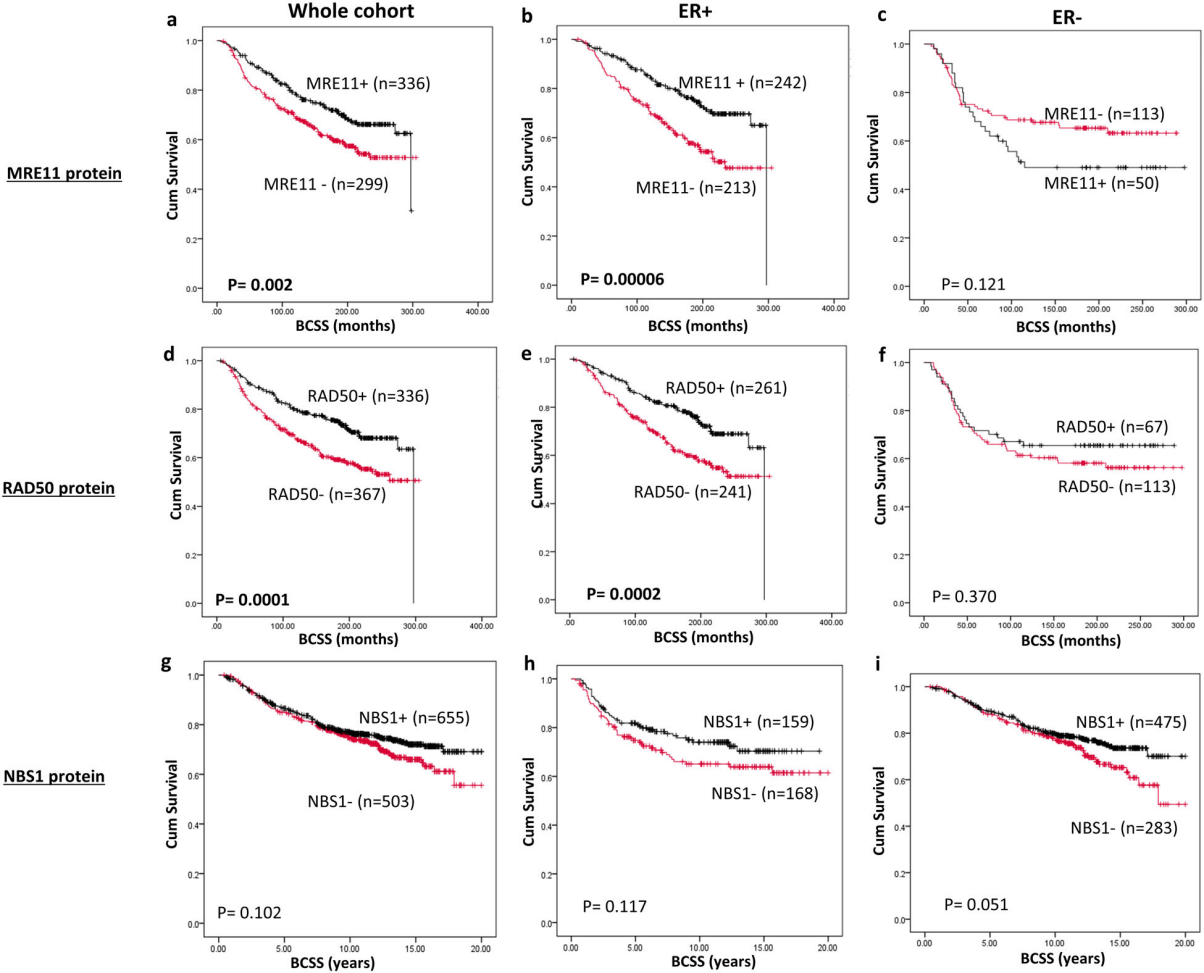
MRN protein expression and survival. **a** Kaplan–Meier curve for MRE11 nuclear protein expression and breast cancer-specific survival (BCSS) in the whole cohort. **b** Kaplan–-Meier curve for MRE11 nuclear protein expression and BCSS in ER+ cohort. **c** Kaplan–Meier curve for MRE11 nuclear protein expression and BCSS in ER− cohort. **d** Kaplan–Meier curve for RAD50 nuclear protein expression and BCSS in the whole cohort. **e** Kaplan–Meier curve for RAD50 nuclear protein expression and BCSS in ER+ cohort. **f** Kaplan–Meier curve for RAD50 nuclear protein expression and BCSS in ER− cohort. **g** Kaplan–Meier curve for NBS1 nuclear protein expression and BCSS in the whole cohort. **h** Kaplan–Meier curve for NBS1 nuclear protein expression and BCSS in ER+ cohort. **i** Kaplan–Meier curve for NBS1 nuclear protein expression and BCSS in ER− cohort.

Low nuclear MRE11 was significantly associated with poor breast cancer-specific survival (BCSS) (*p* = 0.002) (Fig. 2a). In the ER+ cohort, similarly, low nuclear MRE11 was strongly linked with poor BCSS (*p* = 0.00006) (Fig. 2b) but not in ER− tumours (*p* = 0.121) (Fig. 2c). Cytoplasmic expression of MRE11 did not influence survival (Supplementary Fig. 1a–c). Low nuclear RAD50 was significantly associated with poor BCSS (*p* = 0.0001) (Fig. 2d). In the ER+ cohort, similarly, low nuclear RAD50 was also significantly linked with poor BCSS (*p* = 0.0002) (Fig. 2e) but not in ER− tumours (*p* = 0.370) (Fig. 2f). For NBS1, both nuclear (Fig. 2g–I) and cytoplasmic expression did not significantly influence survival (Supplementary Fig. 1D–F). When MRE11, RAD50 and NBS1 were combined, we observed that survival in patients whose tumours had low MRE11/low RAD50/low RAD50 was significantly lower compared to patients whose tumours had high MRE11/high RAD50/high *RAD50* expression (Supplementary Fig. 2). In multivariate analysis, low nuclear RAD50 remained independently associated with BCSS in the whole cohort and in ER+ cohort but not in ER− cohort (Supplementary Table 6). The prognostic significance of RA50 expression was also independent of ER status in breast tumours (Supplementary Table 7). Taken together, the data imply that altered subcellular localization with reduced nuclear expression of MRN influence aggressive histopathological phenotype and survival outcomes.

We then explored if a sub-group of tumours may also have a complete loss of MRN expression [defined as nuclear H-score = 0 and cytoplasmic H-score = 0] by other mechanisms in the breast cancer cohort. As shown in Supplementary Table 8, although rare, we observed a complete loss of MRE11 expression in 14% (90/659) of tumours, complete loss of *RAD50* expression in 5% (40/733) of tumours and complete loss of *NBS1* expression in 9% (109/1166) of tumours. The data suggest that besides altered subcellular localization, complete loss of expression of MRN through mechanisms such as gene deletion, epigenetic silencing, miRNA regulation or post-translational mechanisms may also influence MRN expression and survival. To explore this possibility, we evaluated publicly available genomics datasets.

### *MRE11*, *RAD50* and *NBS1* transcripts and survival

**Fig. 3:**
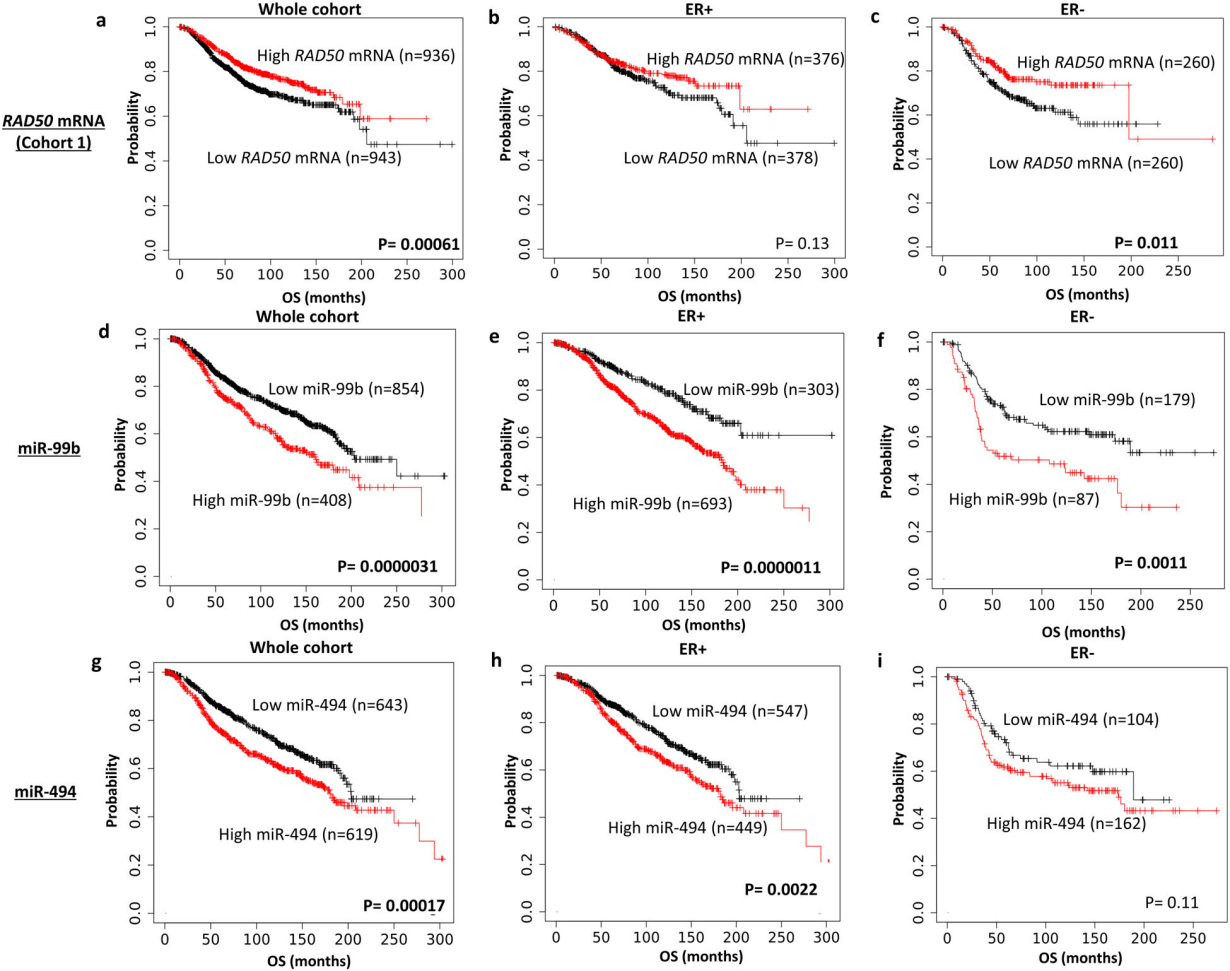
MRN transcripts and survival. **a** Kaplan–-Meier curve for *RAD50* mRNA expression and breast cancer-specific survival (BCSS) in the whole cohort. **b** Kaplan–Meier curve for *RAD50* mRNA expression and BCSS in ER+ cohort. **c** Kaplan–Meier curve for *RAD50* mRNA expression and BCSS in ER− cohort. **d** Kaplan–Meier curve for has-miR-99b expression and BCSS in the whole cohort. **e** Kaplan–Meier curve for has-miR-99b expression and BCSS in ER+ cohort. **f** Kaplan–Meier curves for has-miR-99b expression and BCSS in ER− cohort. **g** Kaplan–Meier curves for has-miR-494 expression and BCSS in the whole cohort. **h** Kaplan–Meier curve for has-miR-494 expression and BCSS in ER+ cohort. **i** Kaplan–Meier curve for has-miR-494 expression and BCSS in ER− cohort.

In a cohort of 1809 breast tumours (cohort 1), low *RAD50 mRNA* was significantly associated with poor survival in the whole cohort (*p* = 0.00061, Fig. 3a) and ER− cohort (*p* = 0.011, Fig. 3c). but not in ER+ cohort (*p* = 0.13, Fig. 3b). In further cohort of 4904 tumours (cohort 2), low *RAD50 mRNA* was significantly associated with poor survival in the whole cohort (*p* = 0.01; Supplementary Figure 3A), ER+ cohort (*p* = 0.03; Supplementary Fig. 3B) but not in ER− cohort (*p* = 0.91; Supplementary Fig. 3C). MRE11 transcript level (Supplementary Fig. 4) or NBS1 transcript level (Supplementary Fig. 5) did not influence survival.

### MRN miRNA regulators and survival

miRNAs are small, highly conserved non-coding RNA molecules that can regulate gene expression. Emerging evidence indicates that *hsa-miR-99b* and *hsa-miR-494* miRNAs^13^ are involved in the regulation of expression of RAD50, MRE11 and NBS1 transcripts. The prognostic significance of hsa-miR-494 and hsa-miR-99b was evaluated in a publicly available miRNA expression dataset from 2178 breast cancer patients. High *hsa-miR-99b* was significantly associated with poor survival in the whole cohort (*p* < 0.0001; Fig. 3d), ER+ cohort (*p* < 0.0001; Fig. 3e) and ER− cohort (*p* = 0.001; Fig. 3f). High *hsa-miR-494* was also significantly linked with poor survival in the whole cohort (*p* = 0.0001; Fig. 3g), ER+ cohort (*p* = 0.0022; Fig. 3h) but not in ER− cohort (*p* = 0.11; Fig. 3I).

Together, the data suggest that the transcriptional regulation of MRN expression in a proportion of tumours may have prognostic significance in breast cancer.

### MRN and genome-wide expression levels

Besides a role in DDR, MRN is also essential for countering oncogene-driven replication stress, dysfunctional telomeres and regulation of innate immune response, thereby contributing to overall cellular homeostasis^1–3^. MRN–ATM axis is also involved in pro-survival signalling, epithelial–mesenchymal transition (EMT), invasion and migration^14,15^. The multifunctional role of the MRN network would imply that either its downregulation or overexpression is likely to have far-reaching consequences at the genome-wide level ultimately promoting or preventing cancer development and prognosis. To explore this hypothesis, we conducted bioinformatics investigations in The Cancer Genome Atlas (TCGA) breast cancer (BRCA) cohort^16,17^.

**Fig. 4:**
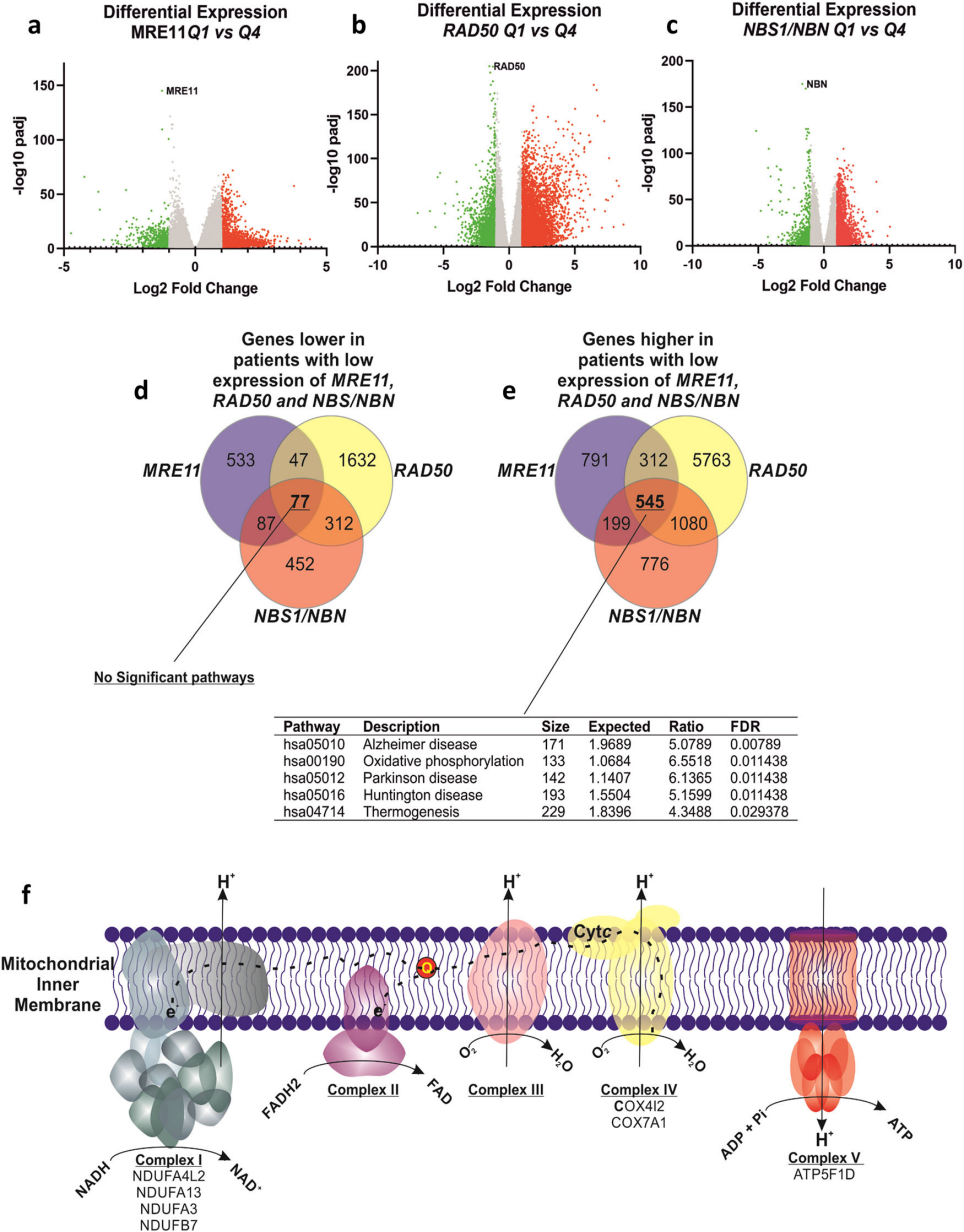
*MRN* and genome-wide alterations. Differentially expressed genes (DEGs) were identified using RNAseq data from the TCGA-BRCA cohort dichotomized on the basis of low (quartile 1) versus high (quartile 4) expression of *MRE11* (**a**)*, RAD50* (**b**) and NBN/NBS1 (**c**). We next identified DEGs that were low (77 genes) or higher (545 genes) in patients with lower expression of MRN components (**d**–**f**) and identified enriched KEGG pathways (**d**, **e**). Pathways related to mitochondrial dysfunction were associated with reduced MRN expression (**f**).

Few coding variants were identified in *MRE11*, *RAD50* and *NBS1*, whereas copy number alternations more commonly affected these loci, particularly for NBS1 (Supplementary Table 9). RNA-sequencing data were obtained from primary female breast cancer specimens (*n* = 1080) from the TGCA breast cancer project and was stratified on the basis of quartile expression of *MRE11*, *RAD50* and *NBS1* and differentially expressed genes (DEGs) identified. This analysis identified 2591 significantly (>2-fold change, *p* adj < 0.05) DEGs between patients with low as compared to high *MRE11* expression, 9768 DEGs between patients with low vs high *RAD50* expression and 3528 DEGs between patients with low vs high *NBS1*/*NBN* (Fig. 4a–c and Supplementary Data 2, 3 and 4). We identified significantly enriched Kyoto Encyclopaedia of Genes and Genomes “KEGG” pathways (Supplementary Tables 10–12), notably in the context of this study including pathways related to pentose metabolism and steroid biosynthesis (hsa00040, hsa00140) associated with *MRE11* (Supplementary Table 10); immune function and surveillance (hsa04060, hsa04657, hsa04650) associated with *RAD50* (Supplementary Table 11); and lipid metabolism, mitochondrial function and inflammatory mediators (hsa04975, hsa00590, hsa04657) associated with *NBS1* expression (Supplementary Table 12). We also examined the chromosomal location distribution of DEGs and identified significantly enriched chromosomal locations for DEGs associated with RAD50 and NBS1 (Supplemental Table 14).

We next identified genes commonly DEGs in patients with low or high expression of *MRE11*, *RAD50* and *NBS1* (Fig. 4d, e). This identified 77 genes with higher expression in patients with high (Q4) expression of *MRE11*, *RAD50* and *NBS1*, whereas there were 545 genes with higher expression in patients with low (Q1) expression of *MRE11*, *RAD50* and *NBS1*. Whereas non-coding genes were abundant among these 77 genes and thus no statistically significant pathways are enriched, key pathways were statistically significantly enriched amongst the 545 genes, including genes involved in oxidative phosphorylation (hsa00190) (Supplemental Table 13). Notably, these 545 genes commonly associated with low MRN were enriched in pathways related to mitochondrial dysfunction, including expression of seven key genes encoding components of complex I (NDUFA3, NDUFA4L2, NDUFA13, NDUFB7), complex IV (COX4I2, COX7A1) and complex V (the ATP synthase component ATP5F1D) (Fig. 4d–f).

## Discussion

The MRN complex is critical for genomic stability^1–3^. Impaired MRN activity can promote a mutator phenotype^18^, thereby leading to malignant transformation. Germline mutations of MRN leading to breast cancer (and other tumours) have been reported albeit extremely rare^7–9^. *MRE11* and *RAD50* are considered as moderate susceptibility genes for breast cancer^19^. Polymorphic variants of MRE11, RAD50 or NBS1 may also increase the risk of development of breast cancer^10,11,20^. In the current study, we have conducted a comprehensive investigation of MRN in sporadic breast cancers. Low MRN is linked with high tumour grade, high mitotic index, ER- and high-risk NPI. Univariate analysis showed an adverse prognostic significance for low nuclear MRE11 and low nuclear *RAD50* expression. In multivariate analysis, low nuclear RAD50 remained independently associated with poor survival. However, a limitation of our study is that patients in this historical cohort (1986–1999) received cyclophosphamide/methotrexate/5-flurouracil (CMF)-based chemotherapy. To further validate the prognostic/predictive significance of MRE11, additional studies in patient who received more modern DNA-damaging chemotherapy will be required. At the transcriptomic level, in both whole cohorts, only low *RAD50* mRNA remained associated with poor survival. Interestingly, in sub-group analysis, in cohort 1 (*n* = 1809) low *RAD50 mRNA* was significantly associated with poor survival in ER− cohort but not in ER+ cohort. In cohort 2 (*n* = 4904), on the other hand, low *RAD50 mRNA* was significantly associated with poor survival in ER + cohort but not in ER− cohort. We speculate that this difference in ER+ and ER− tumours in both cohorts could be related to patient populations receiving different types of chemotherapy and/or endocrine therapy. Nevertheless, a limitation in our study is that we were not able to transcriptionally profile our cohort to further validate protein expression data.

In a mice model of sporadic breast tumorigenesis, disruption of MRE11 (which also disrupts the stability of MRN complex) was shown to promote the progression of mammary hyperplasia into invasive cancer. On the other hand, functional MRE11 prevented cancerous transformation in that study^21^. Our data suggest that low nuclear MRN may impair DNA repair function and promote an aggressive ‘mutator phenotype’ breast cancers. Interestingly, we observed cytoplasmic staining for MRE11 and NBS1, but only nuclear expression status influenced histopathological phenotypes and survival. Although the mechanisms of dysfunctional localization for MRE11 and NBS1 observed here is not yet known, it is possible that cytoplasmic staining may also indicate mitochondrial localization. MRE11 has previously been shown to translocate to mitochondrial following reactive oxygen stress-induced mitochondrial DNA damage^22^. In another study of MRN expression in breast cancers, the authors reported reduced RAD50 (3%), MRE11 (7%) and NBS1 (10%) protein expression, particularly in triple-negative disease, high-grade tumours and in familial breast cancers^23^. RAD50, MRE11 and NBS1 gene sequencing in eight patients from non-BRCA1/2 breast cancer families whose tumours also showed low/loss of RAD50, MRE11 and NBS1 revealed two germline mutations in MRE11, specifically, a missense mutation R202G and a truncating mutation R633STOP (R633X)^23^. Moreover, NBS1 deficiency was associated with poor survival in that study^23^. In the current study, we not only observed low nuclear expression of MRN, but a small proportion of tissue microarray (TMA) cores also had complete loss of MRN expression: MRE11 (14%), RAD50 (5%) and NBS1 (9%). However, a limitation here is that we did not confirm the complete loss of MRN expression in whole tumour sections. Another limitation is that sequence data was unavailable for *MRE11*, *RAD50* or *NBS1* in these specific patients and it was not possible to investigate in patients with familial breast cancers. Nevertheless, our data concur with those reported by Bartkova et al.^23^ demonstrating adverse features in tumour with low MRN levels.

Our study also suggests that the mechanism for low expression of MRN in breast cancers is likely to be multifactorial. MicroRNA regulation, mRNA expression and stability combined with post-translational control could all influence MRN expression^13^. Interestingly, overexpression of *hsa-miR-494* and *hsa-miR-99b* that regulate transcription of MRN was associated with poor clinical outcomes in the current study. At the transcriptomic level, however, only low *RAD50* mRNA expression remained associated with poor survival in the current study. In multivariate analyses, RAD50 protein loss remained independently associated with poor survival. RAD50, which belongs to the structural maintenance of the chromosome protein family, is the largest subunit of MRN^1–3^. The homozygous mutation in the Zn-hook domain of RAD50 is embryonically lethal in mice. The heterozygous mutation can promote liver tumorigenesis. Alteration in the level of RAD50 has been reported in acute myeloid leukaemia, Burkitt lymphoma and endometrial carcinoma^1^. Together, the data imply that RAD50 status may aid prognostic stratification of patients in various cancers including sporadic breast cancers.

Given the multifunctional role of MRN in the maintenance of cellular homeostasis, we speculated that MRN deficiency could have an impact at a genome-wide level and influence aggressive phenotypes in sporadic breast cancer. Pathway analysis of genes associated with MRE11 in breast cancer patients identified significantly enriched pathways associated with steroid hormone biosynthesis, metabolic pathways, ascorbate and aldarate metabolism, complement and coagulation and retinol metabolism. Of interest, the UDP glucronosyltransferase (UGT) family and aldo–keto reductase family were present in these pathways. In cancer, UGTs have altered expression and are linked to drug resistance^24–26^. Furthermore, from the IHC findings that showed both low levels of MRE11 and RAD50 were linked with higher grade tumours, the identification of aldo–keto reductase family in both RAD50 low and MRE11 low tumours highlights a role in tumour progression. AKR1B10 was significantly up-regulated (4-fold change FDR < 0.05) in MRE11 low tumours. ARK1B10 has been shown to be involved tumour progression and metastasis^27^. DEGs associated with high NBS1 identified enriched chromosomal locations including chromosome 8 including the NBS1 locus itself. Genes involved in cell cycle (*CCNB3*, *CCNE2*, *E2F5*), *IDO1*, *IFNG* and *PGR* were all up-regulated, consistent with the clinical parameters identified with high NBS1 levels by IHC. IDO1 has been shown to play a role in breast tumour dormancy, which is an important step in invasive ductal carcinoma^28^. We also identified three significantly enriched pathways (FDR < 0.05), fat digestion and absorption, Ras signalling pathway and arachidonic acid metabolism. *PLA2* genes in arachidonic acid metabolism have been shown to play a role in the mammalian target of rapamycin signalling in breast cancer^29^. While Ras is not commonly mutated in breast cancer, Ras signalling is often up-regulated by other mechanisms^30^. A recent study identified that up-regulation of *H-RAS*, *K-RAS* and *N-RAS* in primary breast cancers was linked to different clinical parameters, with *H-RAS* up-regulation correlating with larger tumour size^31^. We identified up-regulation of *H-RAS* (>2-fold, FDR < 0.05) in tumours that had low *NBS1* expression. Interestingly, low NBS1 protein expression correlated with larger tumour size, suggesting that NBS1 shows a negative correlation with H-RAS signalling. Analysis of DEGs identified in patients with low versus high *RAD50* expression identified 21 significantly enriched KEGG pathways. Notably, the expression of the *ESR1* gene, which encodes ERα was significantly lower in patients with low *RAD50* expression. This is consistent with our findings from IHC that low RAD50 correlated with ER− (FDR < 0.001). IHC also indicated that low RAD50 is associated with poor outcomes/more progressed cancer–lower BCSS, high-grade, high-risk NPI and high mitotic index. In addition to pathways related to inflammation and immune response, JAK-STAT signalling pathway was also significantly over-represented, which indicates potential proliferation, differentiation and anti-apoptotic effects.

Finally, our analysis of DEGs associated with low expression of MRE11, RAD50 and NBS1 identified increased expression of seven genes associated with mitochondrial dysfunction and metabolic reprogramming in cancer. Interestingly, these are nuclear-encoded and five (*ATP5F1D*, *NDUFB7*, *COX7A1*, *NDUFA3*, *NDUFA13*) of the seven genes are encoded on chromosome 19, with *NDUFA4L2* and *COX4I2* encoded on chromosomes 12 and 20, respectively. While chromosome 19 harbours the highest gene density in the human genome^32^, the association of these key nuclear-encoded mitochondrial-associated genes with MRN expression may be functionally related. Thus, although aberrant reduced expression of each MRN component is associated with specific pathways (Supplemental Tables S8–10), reduced expression collectively of MRE11, RAD50 and NBS1 converge with increased expression of key nuclear-encoded mitochondrial proteins (Fig. 4g). Whereas the precise functions of NDUFA3, NDUFA13 (also called GRIM19), COX7A1, COX4I2 and ATP5F1D in oxidative phosphorylation remain somewhat poorly understood, increased expression of NDUFA4L2 is known to drive pro-oncogenic phenotypes in numerous cancer types. NDUFA4L2 is implicated in advanced kidney^33^ and liver^34^ cancers and functions in hypoxia-inducible factor-1α (HIF1α)-induced mitochondrial reprogramming and attenuation of oxidative phosphorylation^35^. This raises the intriguing possibility that aberrant mitochondrial function may contribute to reduced expression of the MRN complex and thereby contribute to poorer outcomes in breast cancers. However, detailed mechanistic studies will be required to explore this hypothesis in detail.

More recently, the MRN complex has also emerged as a target for synthetic lethality and precision medicine^1^. MRE11 deficient in endometrial cancer cells^36^ and colorectal cancer cells^37,38^ have been shown to be sensitive to PARP inhibitors. The data presented in the current clinical study would, therefore, also indicate that MRN-deficient sporadic breast cancers may also be suitable for such a synthetic lethality approach.

## Methods

### MRN complex protein expression

The clinical study was performed in a consecutive series of 1650 patients with primary invasive breast carcinomas who were diagnosed between 1986 and 1999 and entered into the Nottingham Tenovus Primary Breast Carcinoma series. Patient demographics are summarized in Supplementary Table 1. This is a well-characterized series of patients with long-term follow-up that we have investigated in a wide range of biomarker studies^39–42^. All patients were treated in a uniform way in a single institution with standard surgery (mastectomy or wide local excision) with radiotherapy. Prior to 1989, patients did not receive systemic adjuvant treatment (AT). After 1989, AT was scheduled based on prognostic and predictive factor status, including NPI, ER status, and menopausal status. Patients with NPI scores of <3.4 (low risk) did not receive AT. In this historical cohort, for pre-menopausal patients with NPI scores of ≥3.4 (high risk), classical CMF chemotherapy was given; patients with ER+ tumours were also offered endocrine therapy. Postmenopausal patients with NPI scores of ≥3.4 and ER positivity were offered endocrine therapy, while ER− patients received classical CMF chemotherapy. Median follow-up was 111 months (range 1–233 months). Overall survival data were maintained on a prospective basis. BCSS was defined as the number of months from diagnosis to the occurrence of BC-related death. Survival was censored if the patient was still alive at the time of analysis, lost to follow-up, or died from other causes.

Tumour Marker Prognostic Studies (REMARK) criteria, recommended by McShane et al.^43^ were followed throughout this study. Ethical approval was obtained from the Nottingham Research Ethics Committee (C202313). Informed consent was obtained from all human participants.

### TMA and IHC

TMAs were constructed and immunohistochemically profiled for MRE11, RAD50 and NBS1. A set of slides were incubated for 18 h at 4 °C with the primary mouse monoclonal anti-MRE11 antibody (ab214, Abcam), at a dilution of 1:800. A further set of slides were incubated for 18 h at 4 °C with the primary mouse monoclonal anti-RAD50 antibody (Ab489, Abcam), at a dilution of 1:100. A further set of slides were incubated for 18 h at 4 °C with the primary rabbit monoclonal anti-NBS1 antibody (N3162, Sigma), at a dilution of 1:100. We have also profiled a panel of DNA repair markers including those involved in DSB repair (BRCA1, BRCA2, ATM, CHK2, ATR, Chk1, pChk1, RAD51, γH2AX, RPA1, RPA2, DNA-PKcs), RECQ helicases (BLM, WRN, RECQ1, RECQL4, RECQ5), nucleotide excision repair (ERCC1) and base excision repair (SMUG1, APE1, FEN1, PARP1, XRCC1, Pol β). Primary antibodies, clone, source and optimal dilution for each immunohistochemical marker were published previously^40,42,44–55^ and also summarized in Supplementary Table 2.

We validated MRE11, RAD50 and NBS1 antibodies by western blots in a panel of breast cancer cell lines (Fig. 1a). In addition, transient knockdown of MRE11 (manuscript under submission), RAD50 and NBS1 using small interfering RNAs have been performed previously^56,57^ to confirm the validity of the antibody used in the current IHC study. Negative controls for IHC included omission of the primary antibody and IgG-matched serum. Positive control included normal lymphoid (lymph node/spleen) tissue within the TMA. We have previously published the validity of all other markers used in the current study^40,42,44–55^.

### Evaluation of immune staining

The whole field inspection of the core was scored and intensities of the subcellular localization of each marker was identified (nuclear, cytoplasm, cell membrane). Intensities of subcellular compartments were each evaluated and grouped as follows: 0 = no staining, 1 = weak staining, 2 = moderate staining and 3 = strong staining. The percentage of each category was estimated (0–100%). *H*-score (range 0–300) was calculated by multiplying the intensity of staining and percentage staining. Not all cores within the TMA were included for IHC analysis due to missing cores or the absence of tumour cells. The scoring system used for each immunohistochemical marker is summarized in Supplementary Table 2.

### Statistical analyses

Data were performed using SPSS (SPSS, version 17, Chicago, IL). Where appropriate, Pearson’s *χ*^2^, Fisher’s exact, *χ*^2^ for trend, Student’s *t* test and analysis of variance one-way tests were performed using SPSS software (SPSS, version 17, Chicago, IL). Cumulative survival probabilities were estimated using the Kaplan–Meier method. Differences between survival rates were tested for significance using the log-rank test. Multivariate analysis was performed using the Cox hazard model. The proportional hazards assumption was tested using standard log–log plots. Each variable was assessed in the univariate analysis as a continuous and categorical variable and the two models were compared using an appropriate likelihood ratio test. Hazard ratios and 95% confidence intervals were estimated for each variable. Correlation between levels of MRE11, NBS1 and RAD50 protein expressions and other DNA repair biomarkers were generated using RStudio software. The correlation was considered significant at the 0.01 level (two-tailed).

### Transcriptomic and miRNA analyses

Prognostic significance of *MRE11 mRNA* (probe ID 205395_s_at), *RAD50 mRNA* (probe ID _209349_at) and *NBS1 mRNA* (probe ID _202907_s_at) was evaluated in a publicly available microarray dataset from 1809 breast cancer patients (cohort 1)^58^. For additional evaluation, an Affymetric microarray dataset from 4904 breast tumours (cohort 2) was evaluated for *MRE11 mRNA* (median probe data), *RAD50 mRNA* (median probe data) and *NBS1 mRNA* (median probe data)^59^. Prognostic significance miRNAs (*hsa-miR-494* and *hsa-miR-99b*) involved in the regulation of *MRE11*, *RAD50* and *NBS1* mRNA expression was evaluated in a publicly available miRNA expression dataset from 2178 breast cancer patients^60^.

We next analysed the TCGA breast cancer (BRCA) dataset^16,17^ to identify copy number and protein-coding variants affecting *MRE11*, *RAD50* and *NBS1*, and to identify DEGs in breast cancer patients expressing low (quartile 1) and high (quartile 4) levels of *MRE11*, *RAD50* and *NBS1*. To this end, the presence of coding variants (including missense, frameshifts and in-frame deletions) affecting the *MRE11*, *RAD50* and *NBS1* loci were assessed in primary female breast cancer specimens (*n* = 1090) obtained from the TCGA breast cancer dataset. Rare protein-coding variants were identified in *MRE11* (*n* = 7), *RAD50* (*n* = 6) and *NBS1* (*n* = 3). One patient (TCGA-D8-A1J8) harboured mutations in two of the genes (TCGA-D8-A1J8: *MRE11* and *RAD50*).

Separately, RNAseq expression data (HTseq counts) for primary female breast cancer specimens were obtained from the GDC (https://portal.gdc.cancer.gov/) and were dichotomized into low (quartile 1) and high (quartile 4) based on normalized expression (FPKM) of *MRE11*, *RAD50* and *NBS1* obtained from the Xena browser^61^ and DEGs identified using DESeq2^62^. Genes were considered significantly differential expression where fold changes ±2 and FDR < 0.05. Pathway analysis of significant DEGs was conducted using WebGestalt^63^ to interrogate the KEGG database and chromosomal locations. Copy number variants were assessed using the cBioPortal^64^ and expression data in counts format was accessed using the GDC portal^65^.

### Western blot analysis

A panel of breast cancer cells [MCF-7 (ER+, luminal A), ZR-75-1 (ER+, luminal B), SKBR3 (HER2+), MDA-MB-231 (triple-negative)] were harvested and lysed in RIPA buffer (R0278, Sigma) with the addition of protease cocktail inhibitor (P8348, Sigma), phosphatase inhibitor cocktail 2 (P5726, Sigma) and phosphatase inhibitor cocktail 3 (P0044, Sigma) and stored at −20 °C. Protein was quantified using BCA Protein Assay Kit (23227, Thermo Fisher). Membranes were incubated with primary antibodies as follows: anti-MRE11(1:500, ab214, Abcam), anti-RAD50 (1:500, ab89, Abcam) and anti-NBS1 (1:500, N3162, Sigma) at 4 °C overnight. Samples then were washed and incubated with glyceraldehyde 3-phosphate dehydrogenase (1:1000, ab9485) at room temperature for 1 h. Membranes were then washed and incubated with infrared dye-labelled secondary antibodies (Li-Cor) [IRDye 800CW donkey anti-rabbit IgG (926-32213) and IRDye 680CW donkey anti-mouse IgG (926-68072)] at dilution of 1:10,000 for 60 min. The Li-Cor Odyssey Imaging System was utilized for scanning membranes. For quantifying the bands, the Image Studio Lite software (ver 3.1) (Li-Cor, USA) was used. The detected band intensity for the proteins of interest as well as housekeeping gene band intensity was quantified in LI-COR software.

### Reporting summary

Further information on research design is available in the Nature Research Reporting Summary linked to this article.

## Supplementary information

Supplementary Information

Supplementary Data 1

Supplementary Data 2

Supplementary Data 3

Supplementary Data 4

Reporting Summary

## Data Availability

Data supporting the study can be found in the Supplementary information file, and the corresponding author can make any materials available upon request. Aggregate data from the referenced datasets are available from the corresponding author on reasonable request. Primary datasets generated during the study are available in Supplementary Tables 11, 12 and 13. Referenced datasets analysed in the study are described in ‘Methods’ and accession codes are as follows: E-MTAB-365, E-TABM-43, GSE11121, GSE12093, GSE12276, GSE1456, GSE16391, GSE16446, GSE16716, GSE17705, GSE17907, GSE18728, GSE19615, GSE20194, GSE20271, GSE2034, GSE20685, GSE20711, GSE21656, GSE22093, GSE25066, GSE2603, GSE26971, GSE29044, GSE2090, GSE31448, GSE32646, GSE3494, GSE36771, GSE37946, GSE41998, GSE43358, GSE43365, GSE45255, GSE4611, GSE46184, GSE46184, GSE48390, GSE50948, GSE5327, GSE58812, GSE61304, GSE65194, GSE6532, GSE69031, GSE7390, GSE76275, GSE78958, GSE9195, GSE 19783 and GSE 40267.
