# Supplementary material for: Untangling the clinicopathological significance of MRE11-RAD50-NBS1 complex in sporadic breast cancers
Source: NPJ Breast Cancer. 2021 Nov 15;7:143. doi: 10.1038/s41523-021-00350-5 (PMC8593132; doi:10.1038/s41523-021-00350-5)
Supplement: Supplementary file 1 — Supplementary Information [file 41523_2021_350_MOESM1_ESM.pdf]

Supplementary Figures

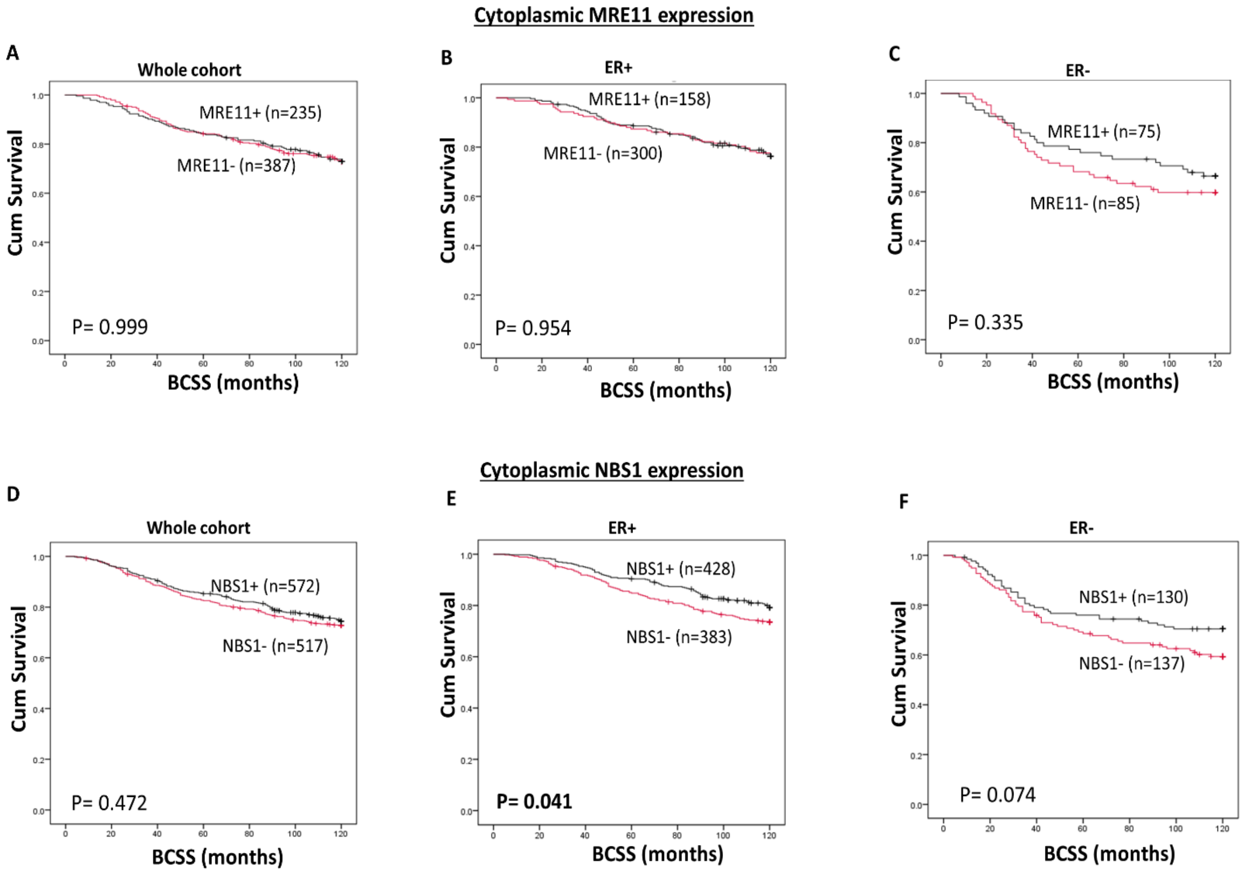

Supplementary Figure 1: A. Kaplan-Meier curve for MRE11 cytoplasmic protein expression and breast cancer specific survival (BCSS) in whole cohort. **B.** Kaplan-Meier curve for MRE11 cytoplasmic protein expression and BCSS in ER+ cohort. **C.** Kaplan-Meier curve for MRE11 cytoplasmic protein expression and BCSS in ER- cohort. **D.** Kaplan-Meier curve for NBS1 cytoplasmic protein expression and BCSS in whole cohort. **E.** Kaplan-Meier curve for NBS1 cytoplasmic protein expression and BCSS in ER+ cohort. **F.** Kaplan-Meier curve for NBS1 cytoplasmic protein expression and BCSS in ER- cohort.

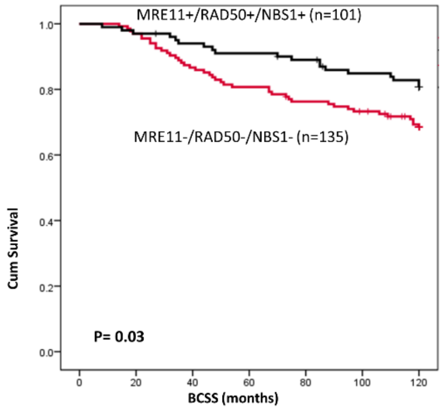

Supplementary Figure 2: Kaplan-Meier curve for low MRN compared to high MRN and BCSS in whole cohort.

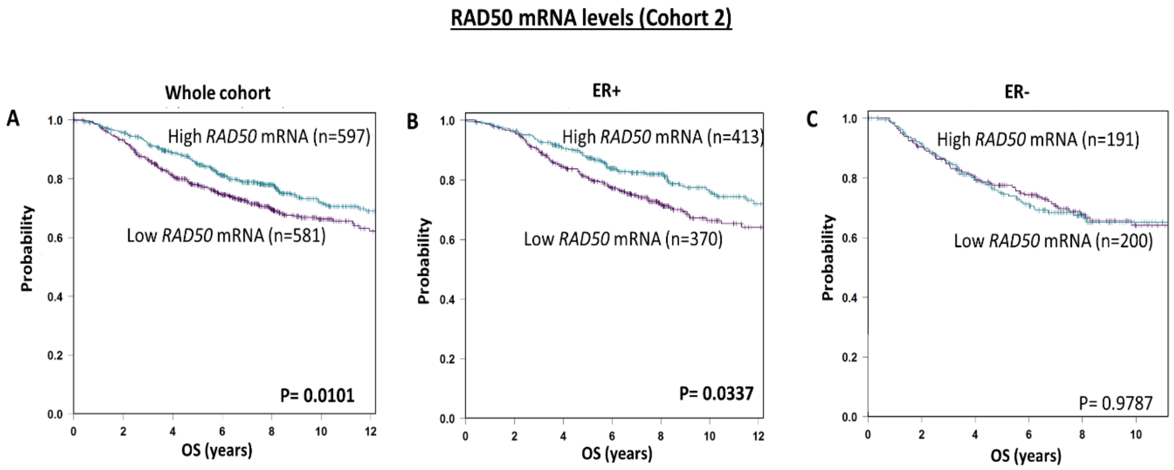

Supplementary Figure 3: A. Kaplan-Meier curve for *RAD50* mRNA expression (cohort 2) and BCSS in whole cohort. **B.** Kaplan-Meier curve for *RAD50* mRNA expression (cohort 2) and BCSS in ER+ cohort. **C.** Kaplan-Meier curve for *RAD50* mRNA expression (cohort 2) and BCSS in ER- cohort.

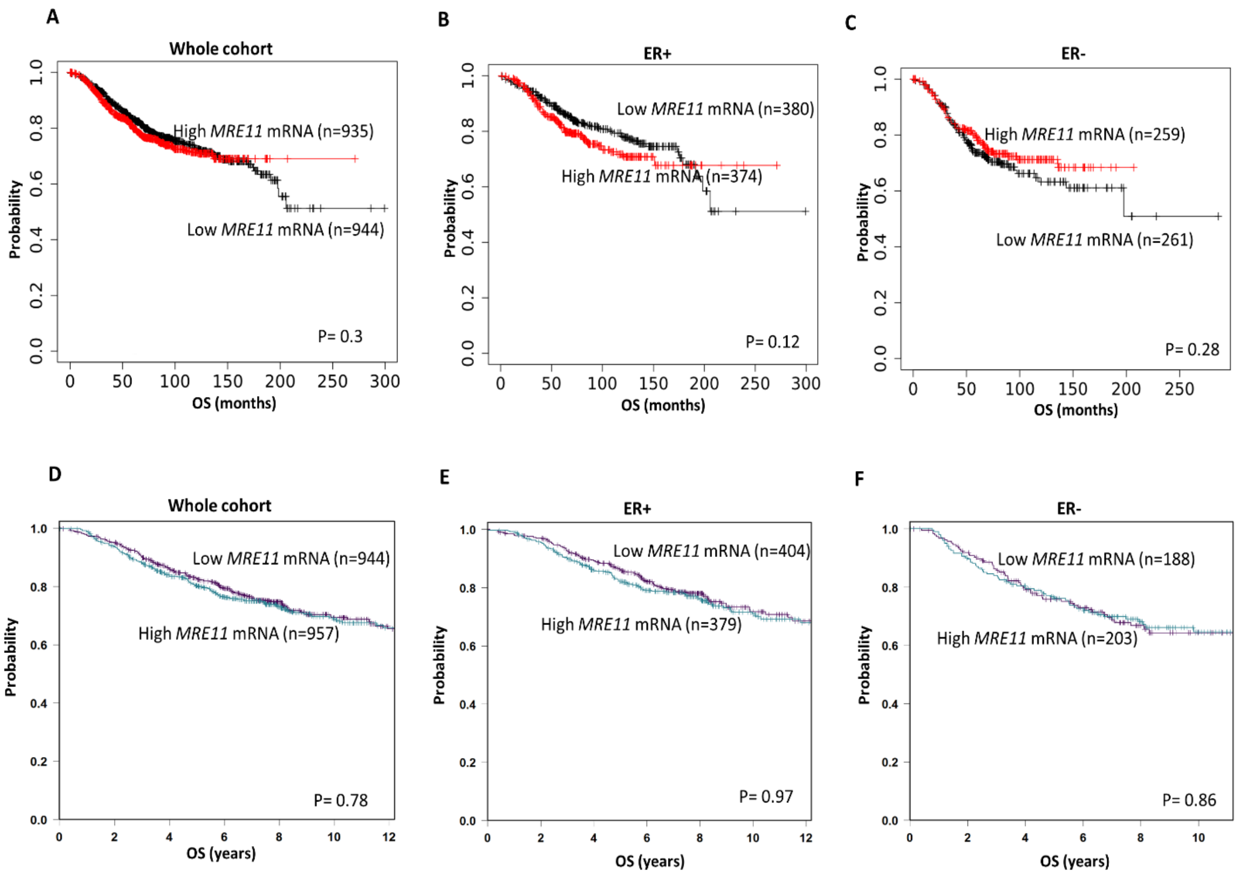

Supplementary Figure 4: A. Kaplan-Meier curve for *MRE11* mRNA expression (cohort 1) and BCSS in whole cohort. **B.** Kaplan-Meier curve for *MRE11* mRNA expression (cohort 1) and BCSS in ER+ cohort. **C.** Kaplan-Meier curve for *MRE11* mRNA expression (cohort 1) and BCSS in ER- cohort. **D.** Kaplan-Meier curve for *MRE11* mRNA expression (cohort 2) and BCSS in whole cohort. **E.** Kaplan-Meier curve for *MRE11* mRNA expression (cohort 2) and BCSS in ER+ cohort. **F.** Kaplan-Meier curve for *MRE11* mRNA expression (cohort 2) and BCSS in ER- cohort.

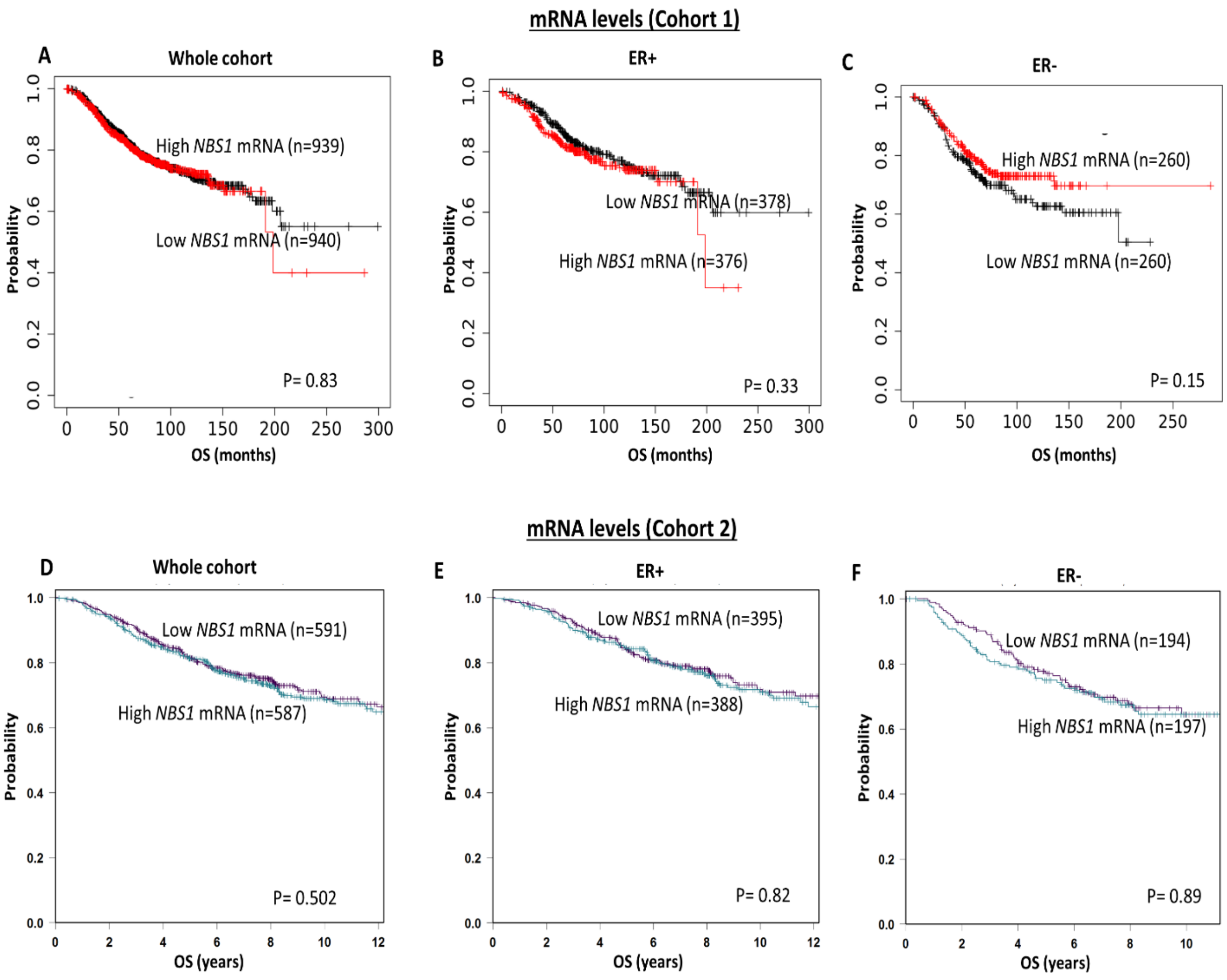

Supplementary Figure 5: A. Kaplan-Meier curve for *NBS1* mRNA expression (cohort 1) and BCSS in whole cohort. **B.** Kaplan-Meier curve for *NBS1* mRNA expression (cohort 1) and BCSS in ER+ cohort. **C.** Kaplan-Meier curve for *NBS1* mRNA expression (cohort 1) and BCSS in ER- cohort. **D.** Kaplan-Meier curve for *NBS1*mRNA expression (cohort 2) and BCSS in whole cohort. **E.** Kaplan-Meier curve for *NBS1* mRNA expression (cohort 2) and BCSS in ER+ cohort. **F.** Kaplan-Meier curve for *NBS1* mRNA expression (cohort 2) and BCSS in ER- cohort.

Supplementary Table S1: Clinicopathological characteristics of Nottingham cohort

Variable	n*	Cases	(%)
**Menopausal status**	1650		
Pre-menopausal		612	(37.0)
postmenopausal		1038	(63.0)
**Tumour Grade (NGS)**	1650		
G1		306	(18.5)
G2		531	(32.2)
G3		813	(49.3)
**Lymph node stage**	1650		
Negative		1056	(64.0)
Positive (1-3 nodes)		486	(29.5)
Positive (>3 nodes)		108	(6.5)
**Tumour size (cm)**	1650		
T1 a + b (≤1.0)		187	(11.0)
T1 c (>1.0 -2.0)		868	(53.0)
T2 (>2.0-5)		579	(35.0)
T3 (>5)		16	(1.0)
**Tumour type**	1650		
IDC-NST		941	(57)
Tubular		349	(21)
ILC		160	(10)
Medullary (typical/atypical)		41	(2.5)
Others		159	(9.5)
**NPI subgroups**	1650		

Excellent PG(2.08-2.40)	Low risk	207	(12.5)
Good PG(2.42-3.40)		331	(20.1)
Moderate I PG(3.42 to 4.4)	High risk	488	(29.6)
Moderate II PG(4.42 to 5.4)		395	(23.9)
Poor PG(5.42 to 6.4)		170	(10.3)
Very poor PG(6.5–6.8)		59	(3.6)
**Survival at 20 years**	1650		
Alive and well		1055	(64.0)
Dead from disease		468	(28.4)
Dead from other causes		127	(7.6)
**Adjuvant systemic therapy (AT)**			
No AT		665	(42.0)
Hormone therapy (HT)		642	(41.0)
Chemotherapy		307	(20.0)
Hormone + chemotherapy		46	(3.0)

* Number of cases for which data were available.

NPI; Nottingham prognostic index, PG; prognostic group

Supplementary Table 2. Antigens, primary antibodies, clone, source, optimal dilution and scoring system used for each immunohistochemical marker.

Antigen	*Antibody*	*Clone*	*Source*	*Antigen Retrieval*	*Dilution / Incubation Time*	*Distribution*	*Scoring system*	*Cut-offs*
**MRE11**	Mouse MAb Anti-MRE11	ab214	Abcam	Citrate pH6	1:800<br>18hours	Nuclear<br>cytoplasmic	H-score<br>H-score	Low nuclear, Median H-score <90<br>Low cyto, Median H-score <10
**RAD50**	Mouse MAb Anti-RAD50	ab89	Abcam	Citrate pH6	1:100<br>18hours	Nuclear	H-score	Low nuclear, Median H-score <100
**NBS1**	Rabbit Anti-NBS1	N3162	Sigma	Citrate pH6	1:100<br>18hours	Nuclear<br>cytoplasmic	H-score<br>H-score	Low nuclear, Median H-score 590<br>Low cyto, Median H-score <70
**BRCA1**	BRCA1	MS110	Calbiochem	Citrate pH6	1:100<br>60 min	Nuclear	% of positive cells	<25% (negative)
**ATM**	Rabbit MAb anti-ATM	Y170	Abcam	Citrate pH6	1:100<br>18 hours	Nuclear	% of positive cells	<25% (negative)
**XRCC1**	Mouse MAb Anti-XRCC1	33-2-5	Thermo-scientific	Citrate pH6	1:200<br>20 min	Nuclear	% of positive cells	≥10% (positive)
**Pol β**	Rabbit anti-polβ	Ab26343	Abcam	Citrate pH6	1:200<br>60 min	Nuclear	H- Score	≥100 (Median H-score, positive)
**BLM**	Rabbit anti BLM	Polyclonal	Novus-Biologicals	Citrate pH6	1:100<br>18 Hours	Nuclear	H- Score	≥50 (Median H-score, positive)
**WRN**	Rabbit Anti-WRN	Polyclonal	Novus Biologicals	Citrate pH6	1:100<br>Overnight (18h)	Nuclear	H-score	Nuclear ≥116(Median H-score High)
**RECQL4**	Rabbit Anti RECQL4	Polyclonal	Novus Biologicals	Citrate pH6	1:1000<br>60 min	Nuclear	H-score	Nuclear ≥215 (Median H-score High)
**CHK2**	Rabbit Anti CHK2	Polyclonal	Abcam	Citrate pH6	1:100<br>60 min	Nuclear	H- Score	≥100 (Median H-score, positive)
**PARP1**	Mouse MAb Anti-PARP1	7D3-6	BD pharmingen	Citrate pH6	1:1000	Nuclear	% of positive cells	≥10% (positive)
**FEN1**	Rabbit anti-FEN1	Polyclonal	Novus Biologicals	Citrate pH6	1:200<br>60 min	Nuclear	H-score	>100 (positive)
**DNA-PKcs**	Mouse MAb Anti-	3H6	Abcam	Citrate pH6	1:1000<br>20 min	Nuclear	H-score	>260 ((Mean H-score, positive)
**ER**	Mouse MAb anti-ER-α	SP1	Dako-Cytomation	Citrate pH6	1:150<br>30 min	Nuclear	Allred score	≥3 (positive)
**ER**	Mouse MAb anti-ER-α	EP1	Dako-Cytomation	Citrate pH6	1:80<br>30 min	Nuclear	% positive cells	≥1% positive
**PR**	Mouse MAb anti-PR	PgR636	Dako-Cytomation	Citrate pH6	1:125<br>30 min	Nuclear	% positive cells	≥1% positive
**HER2**	Rabbit antihuman c-erbB2	Polyclonal	Dako-Cytomation	None	1:400<br>60 min	Membrane	See text	See text
**RPA1**	AB79398	Monoclonal	Abcam	1X Citrate solution	1:100 , 1h room temperature	Nuclear	H-score	112
**RPA2**	AB2175	Monoclonal	Abcam	1X Citrate solution	1:100 , 1h room temperature	Nuclear	H-score	70
**APE1**	Rabbit polyclonal anti-APE1	NB100-101	Novus Biologicals	Citrate pH6	1:500<br>60 min	Nuclear	H-score	≥100 (positive)

**SMUG1**	Goat anti- SMUG1	Polyclonal	Acris Antibody GmbH	Citrate pH6	1/200<br>15 min	Nuclear	H-score	> 35 (positive)
**pChk1**	Rabbit anti-pChk1	Ab58567	Abcam	Citrate pH6	1:140<br>60 min	Nuclear	H-score	≥50 (High)
**ATR**	Mouse MAb Anti-ATR	1E9	Novus Biologicals	Citrate pH6	1:20<br>18 hours	Nuclear	H-score	≥60 (High)
**BRCA2**	Anti-BRCA2	Polyclonal	Sigma	Citrate pH6	1:200<br>Overnight	Nuclear	H-score	>10
**RECQ5**	Rabbit anti RecqL5	Polyclonal	SigmaAldrich	Citrate pH6	1:100<br>60 min	Nuclear	H - Score	≥10 (positive)
**RECQL1**	Rabbit anti-RECQL1	Polyclonal	Bethyl Laboratories	citrate pH 6.0	1:1,000<br>60 min	Nuclear	H-score	=226
**ERCC1**	Mouse anti-ERCC1	4F9	Dako Ltd	citrate pH 6.0	1:150<br>30 min	Nuclear	H-score	≥ 130
**CHK1**	Rabbit anti-CHK1	Polyclonal	Abcam	citrate pH 6.0	1:150<br>60 min	Nuclear	H-score	>30
**RAD51**	Mouse anti-RAD50	Polyclonal	Abcam	citrate pH 6.0	1:70<br>20 min	Nuclear	H-score	Nuclear ≥8 (High)
**γH2AX**	Ab22551(Phospho S139) Mouse	Monoclonal	Abcam	citrate pH 6.0	1:600<br>1h	Nuclear	H-score	≥ 40

Supplementary Table 3. Cytoplasmic expression of MRE11 and NBS1 & clinicopathological features in breast cancer.

	MRE11 (cytoplasmic)				NBS1 (cytoplasmic)		
**VARIABLE**	**Low**	**High**	**P- value**		**Low**	**High**	**P- value**
	**N (%)**	**N (%)**			**N (%)**	**N (%)**	
A) Pathological Parameters							
Tumour Size							
≤2cm	*191 (47)*	*219 (53)*	*0.616*		*254(45)*	*304 (55)*	*0.055*
>2cm	*111 (45)*	*138 (55)*			*311 (51)*	*297 (49)*	
Tumour Stage							
1	*244 (60)*	*155 (62)*	*0.374*		*353 (63)*	*361 (59)*	*0.291*
2	*130 (32)*	*80 (32)*			*161 (29)*	*201 (33)*	
3	*35 (8)*	*14 (6)*			*45 (8)*	*46 (8)*	
NPI							
1(good)	*111 (27)*	*65 (26)*			*166 (30)*	*195 (32)*	
2(moderate)	*223 (54)*	*149 (60)*	*0.257*		*299 (53)*	*313 (52)*	*0.683*
3(poor)	*76 (19)*	*35 (14)*			*93 (17)*	*100 (16)*	
Tumour Grade							
G1	*43 (10)*	*44 (18)*			*82 (15)*	*113 (19)*	
G2	*154 (38)*	*73 (29)*			*187 (33)*	*212 (35)*	
G3	*212 (52)*	*132 (53)*	***0.01***		*290 (52)*	*282 (46)*	*0.098*
Mitotic Index							
M1 (low; mitoses < 10)	*130 (33)*	*67 (28)*			*184 (35)*	*208 (35)*	
M2 (medium; mitoses 10-18)	*86 (22)*	*52 (22)*			*95 (18)*	*121 (20)*	
M3 (high; mitosis >18)	*180 (46)*	*121 (50)*	*0.379*		*253 (47)*	*263 (45)*	*0.451*
Tubule Formation							
1 (>75% of definite tubule)	*12 (3)*	*14 (6)*	*0.061*		*26 (5)*	*39 (7)*	
2 (10%-75% definite tubule)	*123 (31)*	*87 (36)*			*152 (29)*	*236 (40)*	
3 (<10% definite tubule)	*261 (66)*	*139 (58)*			*354 (66)*	*317 (53)*	***<0.001***
Pleomorphism							
1 (small-regular uniform)	*4 (1)*	*10 (4)*			*14 (3)*	*14 (2)*	
2 (Moderate variation)	*147 (37)*	*67 (32)*			*213 (40)*	*218 (37)*	
3 (Marked variation)	*244 (62)*	*154 (64)*	***0.017***		*303 (57)*	*360 (61)*	*0.464*

Tumour Type							
IDC-NST	*342 (83)*	*217 (87)*	**0.003**		*448 (80)*	*522 (86)*	**<0.001**
Tubular Carcinoma	*48 (12)*	*10 (4)*			*77 (14)*	*38 (6)*	
Medullary Carcinoma	*6 (1.5)*	*10 (4)*			*10 (2)*	*12 (2)*	
ILC	*2 (0.5)*	*1 (0)*			*4 (1)*	*1 (0)*	
Others	*12 (3)*	*11 (5)*			*19 (3)*	*35 (6)*	
Lymphovascular Invasion							
No	*256 (63)*	*153 (37)*	0.774		*174 (31)*	*380 (69)*	0.37
Yes	*158 (64)*	*90 (36)*			*217 (36)*	*387 (64)*	
B) Aggressive phenotype							
Her2 overexpression							
No	*344 (86)*	*54 (14)*			*468 (87)*	*67 (13)*	
Yes	*198 (82)*	*44 (18)*	0.116		*495 (86)*	*79 (14)*	0.104
C) Hormone receptors:							
ER/PR Status							
ER^-/PR^-	*85 (21)*	*74 (30)*			*137 (25)*	*132 (23)*	
ER^-/PR^+	*0 (0)*	*0 (0)*			*0 (0)*	*1 (0)*	
ER^+/PR^-	*72(18)*	*31 (13)*			*97 (18)*	*98 (17)*	
ER^+/ER^+	*241 (61)*	*138 (57)*	**0.018**		*309 (57)*	*346 (60)*	0.54

Supplementary Table 4. Associations between MRN complex and other DNA repair protein expression in breast cancers

	MRE11 (Nuclear) PROTEIN EXPRESSION				*RAD50 (Nuclear) PROTEIN EXPRESSION*				*NBS1(Nuclear) PROTEIN EXPRESSION*		
VARIABLE											
	Low	***High***	***P- value***		***Low***	***High***	***P- value***		***Low***	***High***	***P- value***
	N (%)	*N (%)*			*N (%)*	*N (%)*			*N (%)*	*N (%)*	
ATM											
Low	142 (65)	79 (42)			142 (60)	107 (49)			160 (56)	188 (46)	
High	78 (35)	111 (58)	**<0.001**		93 (40)	109 (51)	**0.02**		124 (44)	222 (54)	**0.007**
CHK2											
Low	176 (67)	77 (38)			174 (63)	102 (45)			205 (60)	168 (41)	
High	85 (33)	124 (62)	**<0.001**		104 (37)	126 (55)	**<0.001**		139 (40)	238 (59)	**<0.001**
ATR											
Low	143 (49)	125 (53)			177 (57)	114 (44)			205 (53)	267 (54)	
High	146 (51)	112 (47)	0.457		134 (43)	143 (56)	**0.003**		180 (47)	231 (46)	0.913
CHK1											
Low	221 (86)	155 (76)			226 (85)	183 (79)			266 (82)	298 (76)	
High	37 (14)	48 (24)	**0.011**		41 (15)	49 (21)	0.095		58 (18)	94 (24)	**0.048**
pCHK1											
Low	298 (93)	217 (78)			314 (88)	251 (82)			407 (90)	489 (80)	
High	23 (7)	62 (22)	**<0.001**		42 (12)	54 (18)	**0.032**		44 (10)	119 (20)	**<0.001**
BRCA1											
Low	162 (58)	90 (37)			164 (53)	120 (42)			230 (56)	181 (36)	
High	119 (42)	155 (63)	**<0.001**		146 (47)	168 (58)	**0.006**		180 (44)	327 (64)	**<0.001**
BRCA2											
Low	233 (94)	183 (91)			244 (95)	205 (91)			267 (93)	386 (90)	
High	15 (6)	18 (9)	0.241		14 (5)	21 (9)	0.101		21 (7)	45 (10)	0.152
RAD51											
Low	165 (70)	73 (42)			158 (69)	99 (48)			194 (70)	165 (48)	
High	69 (30)	87 (58)	**<0.001**		72 (31)	105 (52)	**<0.001**		85 (30)	181 (52)	**<0.001**
RPA1											
Low	182 (74)	78 (45)			165 (69)	113 (52)			199 (67)	167 (56)	
High	63 (26)	96 (55)	**<0.001**		74 (31)	104 (48)	**<0.001**		96 (33)	130 (44)	**0.005**
RPA2											
Low	70 (53)	30 (39)			66 (56)	45 (42)			87 (60)	77 (50)	
High	61 (47)	47 (61)	**0.044**		52 (44)	63 (58)	**0.032**		59 (40)	77 (50)	**0.095**
BLM											
Low	83 (29)	44 (19)			105 (34)	40 (15)			115 (30)	109 (23)	
High	201 (71)	190 (81)	**0.006**		202 (66)	222 (85)	**<0.001**		269 (70)	368 (77)	**0.018**
WRN											
Low	87 (49)	80 (46)			113 (54)	71 (39)			114 (42)	160 (46)	

High	91 (51)	94 (54)	0.586		95 (46)	112 (61)	**0.002**		161 (58)	186 (54)	0.233
**RECQ4**											
Low	181 (68)	103 (49)			189 (67)	109 (48)			231 (66)	231 (56)	
High	85 (32)	106 (51)	**<0.001**		93 (33)	120 (52)	**<0.001**		121 (34)	182 (44)	**0.006**
**RECQ5**											
Low	148 (53)	90 (37)			168 (55)	99 (37)			231 (55)	190 (37)	
High	133 (47)	151 (63)	**<0.001**		136 (45)	171 (63)	**<0.001**		192 (45)	328 (63)	**<0.001**
**RECQL1**											
Low	183 (72)	93 (46)			193 (73)	114 (48)			185 (60)	183 (51)	
High	72 (28)	109 (54)	**<0.001**		73 (37)	121 (52)	**<0.001**		124 (40)	176 (49)	**0.021**
**DNA-PKcs**											
Low	71 (27)	20 (9)			67 (25)	30 (12)			89 (25)	41 (10)	
High	190 (73)	199 (91)	**<0.001**		197 (75)	219 (88)	**<0.001**		261 (75)	372 (90)	**<0.001**
**XRCC1**											
Low	73 (26)	17 (8)			69 (23)	27 (11)			86 (24)	52 (11)	
High	212 (74)	203 (92)	**<0.001**		227 (77)	222 (89)	**<0.001**		278 (76)	410 (89)	**<0.001**
**FEN1**											
Low	222 (81)	154 (72)	**0.015**		219 (78)	172 (72)	0.116		274 (79)	287 (66)	**<0.001**
High	51 (19)	60 (28)			63 (22)	68 (28)			72 (21)	150 (34)	
**SMUG**											
Low	87 (33)	33 (16)			85 (31)	47 (19)			117 (34)	65 (16)	
High	179 (67)	1 77 (84)	**<0.001**		191 (69)	197 (81)	**0.003**		224 (66)	346 (84)	**<0.001**
**APE1**											
Low	20 (15)	22 (20)	0.269		24 (19)	28 (18)	0.798		32 (16)	32 (15)	
High	115 (85)	87 (80)			103 (81)	130 (82)			164 (84)	179 (85)	0.748
**Pol β**											
Low	70 (24)	21 (8)			75 (23)	25 (9)			77 (19)	70 (13)	
High	224 (76)	231 (92)	**<0.001**		249 (77)	253 (91)	**<0.001**		324 (81)	471 (87)	**0.009**
**PARP1**											
Low	142 (49)	105 (43)			172 (57)	105 (38)			194 (50)	224 (46)	
High	145 (51)	139 (57)	**0.138**		129 (43)	170 (62)	**<0.001**		196 (50)	264 (54)	0.257
**ERCC1**											
Low	154 (65)	48 (29)			142 (60)	78 (38)			158 (56)	135 (45)	
High	84 (35)	119 (71)	**<0.001**		93 (40)	125 (62)	**<0.001**		125 (44)	163 (55)	**0.011**

Supplemental Table 5. Correlation between levels of MRE11, NBS1, RAD50 protein expressions and other DNA repair biomarkers were generated using RStudio software. Correlation was considered significant at the 0.01 level (2-tailed).

**Low MRE11**	**Low RAD50**	**Low NBS1**
BCRA1 (P<0.001;R=0.291)	ATR (P=0.020;R=0.156)	ATM (P=0.007;R=0.117)
ATM (P<0.001;R=0.197)	BCRA1 (P=0.006;R=0.191)	BLM (P=0.018;R=0.119)
BLM (P=0.006;R=0.235)	BLM (P<0.001;R=0.334)	BCRA1 (P<0.001;R=0.220)
CHK1 (P=0.011;R=0.224)	CHK1 (P=0.007;R=0.120)	CHK1 (P=0.048;R=0.202)
CHK2 (P<0.001;R=0.376)	CHK2 (P<0.001;R=0.248)	CHK2 (P<0.001;R=0.250)
DNA-PKCS (P<0.001;R=0.335)	DNA-PKCS (P<0.001;R=0.278)	DNA-PKCS (P<0.001;R=0.286)
ERCC1 (P<0.001;R=0.433)	ERCC1 (P<0.001;R=0.255)	PCHK1 (P<0.001;R=0.137)
NBS1 (P<0.001;R=0.413)	NBS1 (P<0.001;R=0.271)	POLb (P=0.009;R=0.168)
PCHK1 (P<0.001;R=0.288)	PARP1 (P<0.001;R=0.234)	RAD51 (P<0.001;R=0.210)
POLb (P<0.001;R=0.357)	PCHK1 (P=0.032;R=0.130)	RECQ5 (P<0.001;R=0.238)
RAD50 (P<0.001;R=0.606)	POLb (P<0.001;R=0.289)	RECQL1 (P=0.021;R=0.116)
RAD51 (P<0.001;R=0.334)	RPA2 (P=0.032;R=0.235)	RPA1 (P=0.005;R=0.134)
RECQ4 (P<0.001;R=0.267)	RAD51 (P<0.001;R=0.260)	SMUG (P<0.001;R=0.267)
RECQ5 (P<0.001;R=0.273)	RECQ4 (P<0.001;R=0.263)	γH2AX (P=<0.001;R=0.253)
RECQL1 (P<0.001;R=0.382)	RECQ5 (P<0.001;R=0.217)	
RPA1 (P<0.001;R=0.405)	RECQL1 (P<0.001;R=0.315)	
SMUG1 (P<0.001;R=0.340)	RPA1 (P<0.001;R=0.225)	
γH2AX (P=<0.001;R=0.431)	SMUG1 (P=0.003;R=0.169)	
	γH2AX (P=<0.001;R=0.332)	

Supplementary Table 7. Multivariate analysis

Whole cohort						ER + cohort					ER - cohort			
Variables	Sig.	Exp(B)	95.0% CI for Exp(B)			Sig.	Exp(B)	95.0% CI for Exp(B)			Sig.	Exp(B)	95.0% CI for Exp(B)	
			Lower	Upper				Lower	Upper				Lower	Upper
MRE11(N)	0.415	0.843	0.558	1.272		0.247	0.737	0.44	1.235		0.283	1.437	0.742	2.785
RAD50 (N)	**0.034**	0.648	0.433	0.968		**0.033**	0.578	0.349	0.957		0.655	0.862	0.448	1.656
NBS1 (N))	0.405	1.169	0.809	1.689		0.84	0.954	0.604	1.507		0.128	1.639	0.868	3.095

Supplementary Table 8. Multivariate analysis of RAD50 and ER status in breast cancer

**Whole cohort**				
Variables	Sig.	Exp(B)	95.0% CI for Exp(B)	
			Lower	Upper
RAD50 (N)	**0.002**	0.613	0.453	0.830
ER status	**0.000**	0.558	0.412	**0.758**

Supplementary Table 9. Complete loss of MRN expression in breast cancer cohort

**Marker**	**Number**	**Nuclear H score =0**	**Cytoplasmic H score =0**	**Nuclear & cytoplasmic H score =0**
MRE11	659	96	244	90 (14%)
RAD50	733	40	no cytoplasmic expression	40 (5%)
NBS1	1172	388	241	109 (9%)

Supplemental Table 10: Copy number variants and protein coding variants affecting primary female BCa in the TCGA-BRCA cohort.

Patients (n=1079)	Deletion	Amplification	Protein Coding Variants
MRE11	n=8 (0.7%)	n=10 (0.92%)	E77K;Q218K;R402K;A440T;E451Q;R503C;Q679K
RAD50	n=2	n=4	E152D; E223Q;E305K;V358K;K583Rfs*14;R930I
NBS1	zero	n=145(13.4%)	S213L;H504N/Y;E610K

Supplemental Table 14: Pathways associated with differential *MRE11* expression

Gene Set	Description	Size	Expect	Ratio	FDR
hsa04080	Neuroactive ligand-receptor interaction	277	10.718	2.9856	6.89E-06
hsa05033	Nicotine addiction	40	1.5477	5.1689	0.019575
hsa05034	Alcoholism	180	6.9648	2.5844	0.021414
hsa00040	Pentose and glucuronate interconversions	34	1.3156	5.3209	0.021718
hsa00140	Steroid hormone biosynthesis	60	2.3216	3.8766	0.029392
hsa05322	Systemic lupus erythematosus	133	5.1462	2.7205	0.032705

Supplemental Table 15: Pathways associated with differential *RAD50* expression

Gene Set	Description	Size	Expect	Ratio	FDR
hsa04060	Cytokine-cytokine receptor interaction	294	56.643	2.1892	0
hsa04080	Neuroactive ligand-receptor interaction	277	53.368	1.7801	2.13E-07
hsa04978	Mineral absorption	51	9.8258	2.6461	3.75E-05
hsa05033	Nicotine addiction	40	7.7065	2.8547	4.1E-05
hsa04657	IL-17 signaling pathway	93	17.918	2.1208	7.38E-05
hsa04970	Salivary secretion	90	17.34	2.0185	0.000605
hsa05340	Primary immunodeficiency	37	7.1285	2.5251	0.002362
hsa05332	Graft-versus-host disease	41	7.8992	2.4053	0.002983
hsa04650	Natural killer cell mediated cytotoxicity	131	25.239	1.7037	0.005245
hsa04940	Type I diabetes mellitus	43	8.2845	2.2934	0.005266
hsa04062	Chemokine signaling pathway	189	36.413	1.5379	0.010233
hsa04974	Protein digestion and absorption	90	17.34	1.7878	0.012705
hsa05146	Amoebiasis	96	18.496	1.7301	0.018384
hsa04640	Hematopoietic cell lineage	97	18.688	1.7123	0.020864
hsa04260	Cardiac muscle contraction	78	15.028	1.7967	0.021195
hsa04971	Gastric acid secretion	75	14.45	1.7993	0.023892
hsa04514	Cell adhesion molecules (CAMs)	144	27.743	1.5499	0.026142
hsa04742	Taste transduction	83	15.991	1.6884	0.049501
hsa04630	JAK-STAT signaling pathway	162	31.211	1.4738	0.049501
hsa05330	Allograft rejection	38	7.3212	2.0488	0.049501
hsa05322	Systemic lupus erythematosus	133	25.624	1.522	0.049501

Supplemental Table 16: Pathways associated with differential *NBS1* expression

Gene Set	Description	Size	Expect	Ratio	FDR
hsa04975	Fat digestion and absorption	41	2.6623	3.7561	0.068296
hsa04014	Ras signaling pathway	232	15.065	1.925	0.068296
hsa00590	Arachidonic acid metabolism	63	4.0909	2.9333	0.068296
hsa04950	Maturity onset diabetes of the young	26	1.6883	4.1462	0.085279
hsa04080	Neuroactive ligand-receptor interaction	277	17.987	1.7235	0.12805
hsa04657	IL-17 signaling pathway	93	6.039	2.3183	0.1384

Supplemental Table 17: Pathways associated genes more abundantly expressed in patients with low *MRE11, RAD50* and *NBS1*

Gene Set	Description	Size	Expect	Ratio	FDR
hsa05010	Alzheimer disease	171	1.3508	7.4031	0.00023859
hsa00190	Oxidative phosphorylation	133	1.0506	6.6628	0.010134
hsa05012	Parkinson disease	142	1.1217	6.2405	0.010134
hsa05016	Huntington disease	193	1.5246	5.2474	0.010134
hsa04714	Thermogenesis	229	1.8089	4.4225	0.02614

Supplemental Table 18: Significantly enriched chromosomal locations of DEGs associated with *MRE11, RAD50* or *NBS* expression

Location *MRE11 DEGs*	Size	Expect	Ratio	FDR	Associated Genes
1q21.3	163	7.1158	3.0917	0.002806	*HORMAD1;FLG2;CRNN;S100A8;S100A1;LCE3D SPRR2D;S100A12;SPRR1B;CRCT1; LCE5A;LCE2A LCE1B;LCE1C;C1orf68;LOR;PRR9;SPRR2E;KPRP; LINC01527;SPRR2A;SPRR2F*
9q34.3	128	5.5878	3.0423	0.021549	*PHPT1;LHX3;EDF1;NPDC1;PAXX;FCN2;LCN1;CLIC3;AGPAT2;EGFL7;MRPL41;LRRC26;NOXA1;ENTPD8;CYSRT1;COL5A1-AS1;AJM1*
2q37.3	83	3.6234	3.5878	0.021549	*PRLH;LOC93463;KIF1A;RAMP1;COPS9;MAB21L4; AGXT;AQP12B;MIR149;LOC285095;LOC100130449;LINC01238;MIR2467*

Location *RAD50 DEGs*	Size	Expect	Ratio	FDR	Associated Genes
4q13.3	57	10.931	2.7445	1.0314E-06	*SLC4A4;CXCL2;SULT1E1;ODAM;SMR3A;PF4V1;CXCL6;CSN1S1;STATH;HTN1;CSN2;UGT2B4;ADAMTS3;ALB;CXCL3;CXCL5;PPBP;PF4;CXCL1;CXCL8;SMR3B;CSN3;SULT1B1;AMBN;FDCSP;EPGN;PRR27;AMTN;HTN3;LOC100507388*
22q11.23	51	9.7802	2.2494	0.0027665	*DERL3;DDT;DDTL;SUSD2;UPB1;GGT1;CRYBB3;CRYBB2P1;VPREB3;POM121L9P;IGLL1;LRRC75B;FAM230I;PIWIL3;TMEM211;CES5AP1;BCRP3;FBXW4P1;LINC01659;MIF;CRYBB2;CHCHD10*
17q24.3	21	4.0271	2.9798	0.0043655	*KCNJ16;LINC02097;LINC00511;LINC00673;ROCR SOX9-AS1;LOC102723517;LINC01497;LINC01152 CASC17;LINC02003;KCNJ2-AS1*
1q21.3	163	31.258	1.6316	0.004841	*ANXA9;HORMAD1;FLG2;CRNN;S100A8;S100A7 EFNA3;CELF3;SPRR2G;PGLYRP3;S100A1;CHRNB2 TNFAIP8L2;SCNM1;TNFAIP8L2-SCNM1;S100A11 LCE3D;IVL;SPRR3;SPRR2D;PGLYRP4;S100A9;S100A12;TDRD10;SPRR1B;CRCT1;TCHHL1;SPRR4;S100A7A;LCE3A;LCE5A;LCE1A;LCE2A;S100A3;S100A4; S100A5;LCE1B;S100A2;LCE1C;S100A10;S100A6 C1orf68;LOR;PRR9;SPRR2E;KPRP;LINC01527;C2CD4D;SPRR2A;EFNA4;SPRR2F*
10p15.1	37	7.0954	2.3959	0.0062206	*PRKCQ;IL2RA;IL15RA;AKR1C2;AKR1E2;CALML3;CALML5;AKR1C1;LOC101928051;LINC00703;LINC00705;LINC02677;MANCR;LOC105376384;LOC101927964;PRKCQ-AS1;MIR3155A*
21q22.3	127	24.355	1.6835	0.0086412	*COL6A2;DNMT3L;TFF2;UBASH3A;PDE9A;CBS;CBSL;PDXK;LOC105372824;CSTB;RRP1;GATD3A;GATD3B;AIRE;FAM207A;FTCD;S100B; UMODL1;LINC00334;RIPK4;PCBP3;UMODL1-AS1;LINC00319;LOC100129027;KRTAP10-4;LOC105372842;LINC01678;LINC01671;LOC101928284;ITGB2-AS1;LRRC3-DT;LOC105372833;LINC01694;LINC00163*

					LINC00479;FRGCA;LOC106780825;ZNF295-AS1;LINC00316;LINC01679;CBSL;PICSAR
19q13.2	161	30.875	1.5871	0.011199	*IFNL4;FBXO17;MIA-RAB4B;LOC105372397;CCER2 MIA;CYP2B7P;CYP2A6;LOC105372404;LGALS7;LGALS7B;CYP2A7;CYP2A13;CYP2F1;CYP2B6;IFNL3;RYR1;CXCL17;NCCRP1;SELENOV;ACP7;IFNL2;IFNL1 SYCN;LGALS7;LGALS7B;LGALS4;CEACAM3;PPP1R14A;TMEM145;SPTBN4;GMFG;CYP2G1P;MRPS12;LRFN1;CEACAM8;CNFN;ATP1A3;RABAC1;CEACAM5;RPS19;CD79A;CEACAM4;CLC FBL;RPS16;NFKBIB;MAP4K1;DLL3;SNRPA;POU2F2*
12q13.13	111	21.286	1.6912	0.016006	*CISTR;LINC00592;LOC102724050;KRT73-AS1;HOXC-AS3;KRT81;KRT6A;KRT77;KRT3;KRT5;KRT79;KRT6B;KRT76;;KRT2GTSF1;KRT83;KRT72;KRT74;KRT4;KRT6C;KRT75;KRT86KRT78;SP7;;SOAT2IGFBP6;KRT1;ANKRD33;KRT82;KRT84;GRASP;KRT71;GPR84;KRT7;KRT85;SLC4A8;*
Xq28	152	29.149	1.5781	0.017138	*H2AFB1;LCA10;CSAG3;CSAG2;MAGEA2;MAGEA9B;MAGEA10-MAGEA5;CCNQ;LOC105373383;CSAG4;PNMA6A;MAGEA4-AS1;MAGEA3;PNMA6E;MAGEA12;HAUS7;CSAG1;L1CAM;MAGEA6;LAGE3;MAGEA11;SRPK3 PNMA3;TREX2;MAGEA2B;BGN;SSR4;PASD1;AFF2 RAB39B;MAGEA4;DUSP9;PRRG3;AVPR2;CTAG2;MAGEA10;MAGEA9;GABRE;GPR50;RENBP;NAA10 ARHGAP4;ATP2B3;MAMLD1;GABRA3;TKTL1*
11p15.1	56	10.739	1.9555	0.026216	*ABCC8;USH1C;PIK3C2A;DBX1;MYOD1;KCNC1;TPH1;SAA2;SAA4;UEVLD;NELL1;SAA3P;LDHAL6A;ANO5;SAA1;MRGPRX3;MRGPRX2;OTOG;NCR3LG1;NAV2-AS5;SAA2-SAA4*
6q25.1	33	6.3284	2.2123	0.039744	*ESR1;ULBP1;MTHFD1L;CCDC170;PLEKHG1;ULBP2;ULBP3;ARMT1;RAET1L;PPP1R14C;RAET1G;LOC105378052;RAET1K;TAB2*
18q21.33	20	3.8354	2.6073	0.039744	*SERPINB3;SERPINB7;SERPINB12;BCL2;SERPINB2;SERPINB13;SERPINB11;SERPINB4;SERPINB5;SERPINB10*
3q28	20	3.8354	2.6073	0.039744	*P3H2;CLDN16;CLDN1;TPRG1;OSTN;GMNC;LPP-AS1;P3H2-AS1;TPRG1-AS2;OSTN-AS1*

**Location NBS1/NBN *DEGs***	**Size**	**Expect**	**Ratio**	**FDR**	**Associated Genes**
5q31.1	58	2.310481	5.626534	0.000278	*RAD50;KIF3A;SOWAHA;SLC25A48;FSTL4;JADE2;GDF9;AFF4;IL9;SMAD5;SLC22A5;CXCL14;FNIP1*
5q15	23	0.916225	6.548608	0.023871	*FAM81B;RHOBTB3;KIAA0825;LNPEP;PCSK1;TTC37*
5q11.2	33	1.314584	5.324879	0.023871	*RAB3C;MIER3;CDC20B;MCIDAS;IL6ST;ITGA2;MAP3K1*

5q13.2	33	1.314584	5.324879	0.023871	*OCLN;MARVELD2;CCDC125;BDP1;MCCC2;SERF1A;CARTPT*
5p13.2	29	1.15524	6.059345	0.017046	*CAPSL;TTC23L;RANBP3L;PRLR;AGXT2;SPEF2;LMBRD2*
8p22	21	0.836553	7.172285	0.017046	*NAT2;PSD3;ASAH1;PCM1;SLC7A2;NAT1*
11p15.1	42	1.673107	5.379214	0.006686	*MRGPRX2;LDHAL6A;NCR3LG1;KCNC1;NELL1;PIK3C2A;UEVLD;ABCC8;TPH1*
12q21.31	17	0.67721	8.859882	0.006686	*OTOGL;PTPRQ;MYF6;NTS;LRRIQ1;LIN7A*
12q23.2	17	0.67721	7.383235	0.033025	*SLC5A8;ASCL1;MYBPC1;SYCP3;PAH*
