# Supplementary material for: Untangling the clinicopathological significance of MRE11-RAD50-NBS1 complex in sporadic breast cancers
Source: NPJ Breast Cancer. 2021 Nov 15;7:143. doi: 10.1038/s41523-021-00350-5 (PMC8593132; doi:10.1038/s41523-021-00350-5)
Supplement: Supplementary file 6 — Reporting Summary [file 41523_2021_350_MOESM6_ESM.pdf]

nature research

Corresponding author(s): Prof Srinivasan Madhusudan (Manuscritp ID-NPJBCANCER-01343)

Reporting Summary

Nature Research wishes to improve the reproducibility of the work that we publish. This form provides structure for consistency and transparency in reporting. For further information on Nature Research policies, see Authors & Referees and the Editorial Policy Checklist.

Statistical parameters

When statistical analyses are reported, confirm that the following items are present in the relevant location (e.g. figure legend, table legend, main text, or Methods section).

n/a	Confirmed	
☐	☒	The exact sample size (*n*) for each experimental group/condition, given as a discrete number and unit of measurement
☒	☐	An indication of whether measurements were taken from distinct samples or whether the same sample was measured repeatedly
☐	☒	The statistical test(s) used AND whether they are one- or two-sided<br>*Only common tests should be described solely by name; describe more complex techniques in the Methods section.*
☐	☒	A description of all covariates tested
☒	☐	A description of any assumptions or corrections, such as tests of normality and adjustment for multiple comparisons
☐	☒	A full description of the statistics including central tendency (e.g. means) or other basic estimates (e.g. regression coefficient) AND variation (e.g. standard deviation) or associated estimates of uncertainty (e.g. confidence intervals)
☒	☐	For null hypothesis testing, the test statistic (e.g. *F*, *t*, *r*) with confidence intervals, effect sizes, degrees of freedom and *P* value noted<br>*Give P values as exact values whenever suitable.*
☒	☐	For Bayesian analysis, information on the choice of priors and Markov chain Monte Carlo settings
☒	☐	For hierarchical and complex designs, identification of the appropriate level for tests and full reporting of outcomes
☒	☐	Estimates of effect sizes (e.g. Cohen's *d*, Pearson's *r*), indicating how they were calculated
☐	☒	Clearly defined error bars<br>*State explicitly what error bars represent (e.g. SD, SE, CI)*

Our web collection on statistics for biologists may be useful.

Software and code

Policy information about availability of computer code

Data collection	Transcriptomic and miRNA analyses was completed with an online platforms<br>1. http://kmplot.com/analysis/index.php?p=service&cancer=breast<br>2. http://bcgenex.ico.unicancer.fr/BC-GEM/GEM-requete.php<br><br>Detailed bio-informatics was completed using the TCGA breast cancer dataset available at:<br>https://www.cancer.gov/about-nci/organization/ccg/research/structural-genomics/tcga<br><br>RNAseq expression data (HTseq-counts) for primary female breast cancer specimens were obtained from the GDC (https://portal.gdc.cancer.gov/)

Data analysis	Transcriptomic and miRNA analyses was completed with an online platforms<br>1. http://kmplot.com/analysis/index.php?p=service&cancer=breast<br>2. http://bcgenex.ico.unicancer.fr/BC-GEM/GEM-requete.php<br><br>Detailed bio-informatics was completed using the TCGA breast cancer dataset available at:<br>https://www.cancer.gov/about-nci/organization/ccg/research/structural-genomics/tcga<br><br>RNAseq expression data (HTseq-counts) for primary female breast cancer specimens were obtained from the GDC (https://portal.gdc.cancer.gov/)

For manuscripts utilizing custom algorithms or software that are central to the research but not yet described in published literature, software must be made available to editors/reviewers upon request. We strongly encourage code deposition in a community repository (e.g. GitHub). See the Nature Research guidelines for submitting code & software for further information.

Data

Policy information about availability of data

All manuscripts must include a data availability statement. This statement should provide the following information, where applicable:

- Accession codes, unique identifiers, or web links for publicly available datasets
- A list of figures that have associated raw data
- A description of any restrictions on data availability

Provide your data availability statement here.

Field-specific reporting

Please select the best fit for your research. If you are not sure, read the appropriate sections before making your selection.

[x] Life sciences [ ] Behavioural & social sciences [ ] Ecological, evolutionary & environmental sciences

For a reference copy of the document with all sections, see nature.com/authors/policies/ReportingSummary-flat.pdf

Life sciences study design

All studies must disclose on these points even when the disclosure is negative.

Sample size	The clinical study was performed in a consecutive series of 1650 patients with primary invasive breast carcinomas who were diagnosed between 1986 and 1999 and entered into the Nottingham Tenovus Primary Breast Carcinoma series.
Data exclusions	None
Replication	Transcriptomic analysis was performed in test cohort and a validation cohort.<br>All western blots were performed in triplicates
Randomization	Not applicable
Blinding	Not applicable

Reporting for specific materials, systems and methods

Materials & experimental systems

n/a	Involved in the study	
[x]	[ ]	Unique biological materials
[ ]	[x]	Antibodies
[ ]	[x]	Eukaryotic cell lines
[x]	[ ]	Palaeontology
[ ]	[ ]	Animals and other organisms
[ ]	[x]	Human research participants

Methods

n/a	Involved in the study	
[x]	[ ]	ChIP-seq
[x]	[ ]	Flow cytometry
[x]	[ ]	MRI-based neuroimaging

Antibodies

Antibodies used	TMAs were constructed and immunohistochemically profiled for MRE11, RAD50 and NBS1. A set of slides were incubated for 18 hours at 40C with the primary mouse monoclonal anti-MRE11 antibody, (ab214, abcam), at a dilution of 1:800. A further set of slides were incubated for 18 hours at 40C with the primary mouse monoclonal anti-RAD50 antibody (Ab489, Abcam), at a dilution of 1:100. A further set of slides were incubated for 18 hours at 40C with the primary rabbit monoclonal anti-NBS1 antibody (N3162, sigma), at a dilution of 1:100.
Validation	We validated MRE11, RAD50 and NBS1 antibody by western blots in a panel of breast cancer cell lines . In addition, transient knock down of MRE11 (manuscript under submission), RAD50 and NBS1 using siRNAs have been performed previously 30,31 to confirm the validity of the antibody used in the current IHC study. Negative controls for IHC included omission of the primary antibody and IgG-matched serum. Positive control included normal lymphoid (Lymph Node/spleen) tissue within the TMA.

Eukaryotic cell lines

Policy information about cell lines

Cell line source(s)	A panel of breast cancer cells [MCF-7 (ER+, luminal A), ZR-75-1 (ER+, luminal B), SKBR3 (HER2+), MDA-MB-231 (triple negative)] were obtained from ATCC.
Authentication	STR profiling
Mycoplasma contamination	All cell lines were routinely tested for mycoplasma on a 3 monthly basis
Commonly misidentified lines (See ICLAC register)	None

Animals and other organisms

Policy information about studies involving animals; ARRIVE guidelines recommended for reporting animal research

Laboratory animals	na
Wild animals	na
Field-collected samples	*For laboratory work with field-collected samples, describe all relevant parameters such as housing, maintenance, temperature, photoperiod and end-of-experiment protocol OR state that the study did not involve samples collected from the field.*

Human research participants

Policy information about studies involving human research participants

Population characteristics	The clinical study was performed in a consecutive series of 1650 patients with primary invasive breast carcinomas who were diagnosed between 1986 and 1999 and entered into the Nottingham Tenovus Primary Breast Carcinoma series. All patients provided informed consent. Ethical approval was obtained from the Nottingham Research Ethics Committee (C202313).
Recruitment	None
